# Aspergixanthones I–K, New Anti-*Vibrio* Prenylxanthones from the Marine-Derived Fungus *Aspergillus* sp. ZA-01

**DOI:** 10.3390/md16090312

**Published:** 2018-09-04

**Authors:** Ao Zhu, Xing-Wang Zhang, Miao Zhang, Wan Li, Zheng-Yue Ma, Hua-Jie Zhu, Fei Cao

**Affiliations:** 1 Key Laboratory of Pharmaceutical Quality Control of Hebei Province, Key Laboratory of Medicinal Chemistry and Molecular Diagnostics of Education Ministry of China, College of Pharmaceutical Sciences, Hebei University, Baoding 071002, China; 18233235123@163.com (A.Z.); mzhanghbu@163.com (M.Z.); liwanjingmin@163.com (W.L.); mazhengy@126.com (Z.-Y.M.); 2 Shandong Provincial Key Laboratory of Synthetic Biology, CAS Key Laboratory of Biofuels, Qingdao Institute of Bioenergy and Bioprocess Technology, Chinese Academy of Sciences, Qingdao 266101, China; zhangxw@qibebt.ac.cn

**Keywords:** marine-derived fungus, *Aspergillus* sp., prenylxanthone, anti-*Vibrio* activity

## Abstract

Marine-derived fungi are a rich source of structurally diverse metabolites. Fungi produce an array of compounds when grown under different cultivation conditions. In the present work, different media were used to cultivate the fungus *Aspergillus* sp. ZA-01, which was previously studied for the production of bioactive compounds, and three new prenylxanthone derivatives, aspergixanthones I–K (**1**–**3**), and four known analogues (**4**–**7**) were obtained. The absolute configuration of **1** was assigned by ECD experiment and the Mo_2_(AcO)_4_ ICD spectrum of its methanolysis derivative (**1a**). All the compounds (**1**–**7**) were evaluated for their anti-*Vibrio* activities. Aspergixanthone I (**1**) showed the strongest anti-*Vibrio* activity against *Vibrio parahemolyticus* (MIC = 1.56 μM), *Vibrio anguillarum* (MIC = 1.56 μM), and *Vibrio alginolyticus* (MIC = 3.12 μM).

## 1. Introduction

Xanthones, usually obtained from many marine-derived fungi, are a class of secondary metabolites containing a polysubstituted 9*H*-xanthen-9-one skeleton [1]. They are described as “privileged structures” in the field of modern medicine [2], due to their pronounced pharmacological activities, including antibacterial [3], antifungal [4], cancer chemopreventive [5,6], and cytotoxic activities [7]. Among them, prenylxanthones have been mainly isolated from the fungi of the genus *Aspergillus*/*Emericella* [8,9,10]. The first prenylxanthone derivative, tajixanthone, was isolated from the fungus *Aspergillus variecolor* by Chexal et al. in 1974 [11]. Since then, about 20 bioactive prenylxanthone analogues have been obtained, including ruguloxanthones A–C [12] and emerixanthones A–D [10].

**Figure 1:**
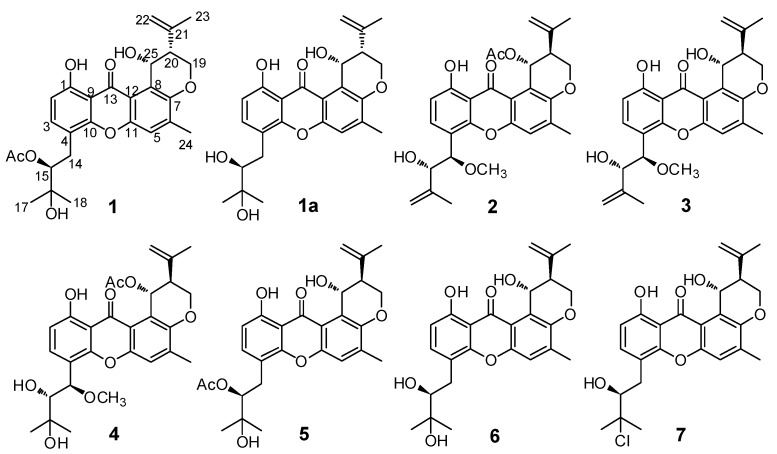
Chemical structures of **1**–**7**.

In our previous investigation on the marine-derived fungus *Aspergillus* sp. ZA-01, several new cytotoxic 14,15-hydroxylated prenylxanthones, aspergixanthones A–H were obtained from cultures grown in rice solid medium [9]. Fungal strains are reported to produce an array of constituents when grown under different cultivation conditions [13], including variations in the composition of culture medium, period of cultivation, the pH, and the temperature. Different HPLC-UV profiles of the EtOAc extract were obtained when fermentation of strain ZA-01 was carried out using a shaken Czapek-Dox medium. Further systematic chemical exploration of this extract led to the isolation of three new prenylxanthone derivatives, aspergixanthones I–K (**1**–**3**), and four known analogues: aspergixanthone A (**4**) [9], 15-acetyl tajixanthone hydrate (**5**) [14], tajixanthone hydrate (**6**) [15], and 16-chlorotajixanthone (**7**) [15] (Figure 1). Herein, we report the isolation, structure elucidation, absolute configurations, and anti-*Vibrio* activities of these compounds (**1**–**7**).

## 2. Results

**Figure 2:**
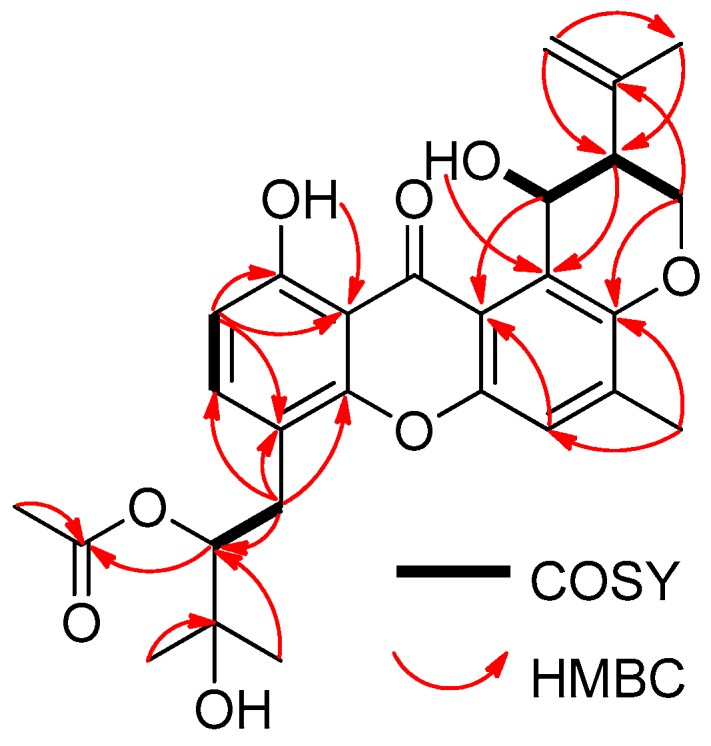
COSY and key HMBC correlations of **1**.

Aspergixanthone I (**1**) was obtained as a yellow powder, which showed five maximum UV absorbance bands at 228, 242, 264, 285, and 385 nm, indicating a prenylxanthone nucleus for **1** [8,9,10]. The molecular formula of C_27_H_30_O_8_ for **1** was deduced from the molecular ion peak [M + Na]^+^ at *m/z* 505.1827 (calculated (calcd.) for C_27_H_30_O_8_Na, 505.1833) in positive HRESIMS, which corresponded to 13 degrees of unsaturation. The ^1^H NMR and ^13^C NMR data of **1** (Table 1 and Table 2), showed the presence of four methyl signals (*δ*_H_ 2.38 (3H, s, H-24), 1.86 (3H, s, H-23), 1.38 (3H, s, H-18), and 1.34 (3H, s, H-17); *δ*_C_ 26.9 (C-17), 25.3 (C-18), 22.5 (C-23), and 17.4 (C-24)), one oxygen-bearing methylene signal (*δ*_H_ 4.46 (1H, brd, *J* = 10.8 Hz, H-19a) and 4.35 (1H, dd, *J* = 12.0, 10.8 Hz, H-19b); *δ*_C_ 64.1 (C-19)), three aromatic methine signals (*δ*_H_ 7.41 (1H, d, *J* = 8.4 Hz, H-3), 7.29 (1H, s, H-5), and 6.71 (1H, d, *J* = 8.4 Hz, H-2); *δ*_C_ 137.9 (C-3), 119.5 (C-5), and 109.5 (C-2)), and one keto carbonyl signal (*δ*_C_ 184.5 (C-13)), confirming the prenylxanthone skeleton of **1** [8,9,10]. In fact, the structure of **1** was closely related to that of compound epitajixanthone hydrate, a prenylxanthone derivative that was previously isolated from the endophytic fungus *Emericella* sp. XL029 [8]. Additional signals for an acetoxy (*δ*_H_ 1.99 (3H, s); *δ*_C_ 170.4 and 20.7) were present in the NMR spectra of **1**, implicating an epitajixanthone hydrate analogue bearing an additional acetoxy group for **1**. The position of this 15-OAc unit was deduced from the proton spin system of H-14/H-15 from the ^1^H-^1^H COSY spectrum (Figure S4), and the long-range couplings of H-15/15-COCH_3_ and H-18/C-15 in the HMBC spectrum (Figure S5) of **1** (Figure 2). Thus, **1** was the 15-acetyl derivative of epitajixanthone hydrate.

**Figure 3:**
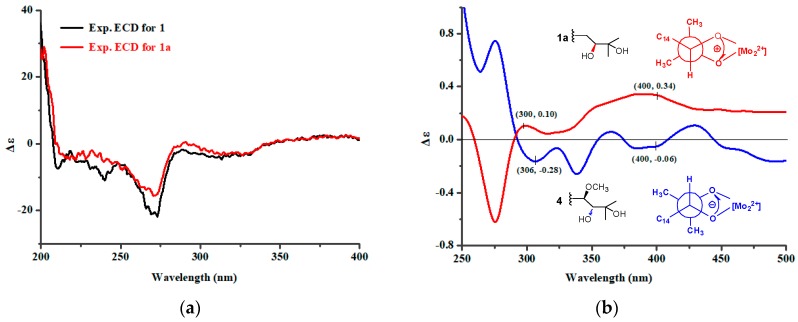
(**a**) Experimental ECD spectra for **1** and **1a**; (**b**) ICD spectra of Mo-complexes of **1a** (red) and **4** (blue) recorded in DMSO.

In order to define the relative and absolute configurations of **1**, the methanolysis derivative of **1** (**1a**) was prepared using K_2_CO_3_ in anhydrous MeOH. The NMR data of **1a** were identical to those of epitajixanthone hydrate, suggesting that **1a** and epitajixanthone hydrate were the same compound, and that **1** and epitajixanthone hydrate had the same stereoconfiguration. This deduction was verified by the NOESY correlation (Figure S6) between H-20 and H-25 in **1**, and the positive specific rotation value ([α]${}_{20}^{D}$ = +42.5 (*c* 0.10, MeOH)) of **1** [8,9]. Additionally, the same ECD cotton effects of **1** and epitajixanthone hydrate (**1a**) (Figure 3a) indicated that **1** had the same stereoconfiguration as epitajixanthone hydrate (**1a**), whose relative configuration was determined using crystal data (Mo *K*α radiation) [8]. To assign the absolute configuration of **1a**, the dimolybdenum tetraacetate (Mo_2_(AcO)_4_) ICD procedure (Snatzke’s method) was used. The positive ICD cotton effects at 300 (0.10) and 400 (0.34) nm of **1** gave the Newman form of the Mo-complexes of **1** (Figure 3b), which showed a clockwise rotation, and suggested a 15*S* configuration for **1a** [16,17]. Based on the above data analysis, the absolute configuration of **1** could be defined as 15*S*,20*R*,25*R*.

**Figure 4:**
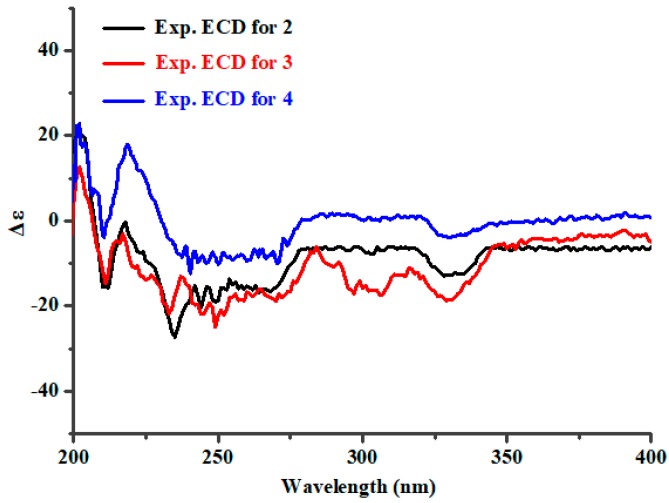
Experimental ECD spectra for **2**–**4**.

Aspergixanthone J (**2**) showed an [M + Na]^+^ ion peak at *m/z* 517.1826, indicating a molecular formula of C_28_H_30_O_8_. The NMR data of **2** (Table 1 and Table 2) closely resembled those of aspergixanthone A (**4**) [9], except for the signals for the 17-Me in aspergixanthone A (**4**) being replaced by those for an olefinic methylene (*δ*_H_ 4.65 (1H, brs, H-17a) and 4.62 (1H, brs, H-17b); *δ*_C_ 114.8 (C-17)), indicating the presence of a double bond between C-16 and C-17 in **2**. Analysis of HMBC correlations from H-17 to C-15/C-16/C-18 demonstrated the elucidation of the plane structure of **2**. The NOESY correlations (Figure S16), the coupling constants, the negative specific rotation value of **2**, and the similarity of the ECD spectra of **2** and **4** (Figure 4) suggested that **2** had the same absolute configuration as **4**, which was previously assigned as 14*R*,15*R*,20*S*,25*R* by a combined analysis of ECD, ORD, and VCD methods [9]. In particular, the absolute configuration at C-15 in **4** was demonstrated to be *R*, using Snatzke’s method (Figure 3b), unambiguously, which was opposite to **1**.

Aspergixanthone K (**3**) was determined to have a molecular formula of C_26_H_28_O_7_ using HRESIMS analysis. The 1D and 2D NMR data of **3** (Table 1 and Table 2) revealed that **3** represents a structural analogue of **2**, but it is missing the acetoxy group at C-25. The unambiguous ^1^H-^1^H COSY cross-peaks of 25-OH/H-25/H-20/H-19 confirmed the postulated 25-deacetylation homologue of **2**. Similar NOESY correlations (Figure S23) and ECD spectra of **2** and **3** implied that they had the same stereoconfigurations.

Prenylxanthone derivatives (**1**–**7**) are a class of bioactive natural compounds that belong to the family of naturally occurring xanthones [1]. These prenylxanthones with a C-4 terpenoid-derived side chain were mainly isolated from fungi of the genus *Aspergillus*/*Emericella* [8,9,10]. It was an interesting and challenging task to define the stereoconfigurations of the C-4 side chain for these prenylxanthone derivatives. In particular, the absolute configuration at C-15 in prenylxanthone derivatives was often assigned by comparison of the specific rotation with that of previous reports [8,14], which was inappropriate, since the absolute configuration of C-15 had nothing to do with specific rotation [9]. In this work, two possible absolute configurations for C-15 were present in different prenylxanthone derivatives, which were assigned using Snatzke′s method.

*Vibrio* spp., such as *Vibrio anguillarum*, *Vibrio parahemolyticus*, and *Vibrio alginolyticus*, are a class of Gram-negative halophilic bacteria that usually occur in marine and coastal environments throughout the world, which could lead to vibriosis in crustaceans and cause serious damage to mariculture production [18,19]. However, there is no effective vaccine to prevent vibriosis due to lacking adaptive immunity in crustacean species. In the past few decades, searching for anti-*Vibrio* agents from marine-derived fungi for controlling vibriosis has become one of the research trends. Therefore, the anti-*Vibrio* activities against *V.* *parahemolyticus*, *V. anguillarum*, and *V.* *alginolyticus* of **1**–**7** were tested. All of the compounds (**1**–**7**) showed anti-*Vibrio* activities to three pathogenic *Vibrio* spp., with MIC values between 1.56 and 25.0 μM (Table 3). Among them, aspergixanthone I (**1**) exhibited the strongest anti-*Vibrio* activity, indicating that the propenyl at C-20 with *α*-stereoconfiguration may play an important role for the anti-*Vibrio* activity.

## 3. Experimental Section

### 3.1. General Experimental Procedures

Specific rotations: AA-55 series polarimeter (Optical Activity Ltd., Cambridgeshire, UK). UV spectra: a multiskan go microplate spectrophotometer (Thermo Scientific Co., Waltham, MA, USA). Electronic circular dichroism curves: J-815 spectropolarimeter (JASCO Electric Co., Ltd., Tokyo, Japan). IR spectra: Nicolet NEXUS 470 spectrophotometer (Thermo Electron Co., Madison, WI, USA) using KBr pellets. 1D and 2D NMR spectra: Bruker AVIII 600 MHz NMR spectrometer (Bruker BioSpin GmbH Co., Rheinstetten, Germany), using the residual solvent resonance as an internal standard. Semi-preparative HPLC: Shimadzu LC-20AT system with a SPD-M20A photodiode array detector (Shimadzu Co., Kyoto, Japan), and Waters RP-18 (XBridge OBD, 5 μm, 10 mm × 250 mm).

### 3.2. Isolation of the Fungal Material

The fungus *Aspergillus* sp. ZA-01 has been previously described [9]. Liquid fermentation of the fungus *Aspergillus* sp. ZA-01 using shaken Czapek-Dox medium (150 rpm, 30 L, 1 L Erlenmeyer flasks each containing 500 mL of culture broth) was performed at 30 °C for 14 days. The culture was filtered to separate the culture broth from the mycelia and was repeatedly extracted using EtOAc (10 L) at room temperature six times, which yielded a crude extract (3.2 g). The extract was then chromatographed on a silica gel column using a stepwise gradient of petroleum ether (PE)/EtOAc (100:0 to 0:100) to produce six fractions: Fr.1–Fr.6. Fr.3 was further purified by silica gel CC (PE:EtOAc = 2:1), Sephadex LH-20 (CH_2_Cl_2_:MeOH = 1:1), and preparative HPLC using a C_18_ column (CH_3_OH:H_2_O = 73:27) to provide **1** (5.2 mg, *t*_R_ 20.5 min), **2** (2.0 mg, *t*_R_ 28.4 min), **3** (2.3 mg, *t*_R_ 25.1 min), **4** (4.6 mg, *t*_R_ 13.6 min), **5** (6.2 mg, *t*_R_ 16.2 min), **6** (5.0 mg, *t*_R_ 11.0 min), and **7** (4.1 mg, *t*_R_ 22.3 min).

Aspergixanthone I (**1**): yellow, amorphous powder; [α]${}_{20}^{D}$ = +42.5 (*c* 0.10, MeOH); UV (MeOH) *λ*_max_ (log *ε*) 230 (4.5), 243 (4.0), 266 (4.7), 285 (2.1), 382 (1.9) nm; IR (KBr) *ν*_max_ 3451, 2930, 2356, 1637, 1593, 1462, 1257, 1081, 903 cm^–1^; NMR data, see Table 1 and Table 2; HRESIMS *m/z* 505.1827 [M + Na]^+^, (calcd. for C_27_H_30_O_8_Na, 505.1833).

Aspergixanthone J (**2**): yellow, amorphous powder; [α]${}_{20}^{D}$ = −78.2 (*c* 0.1, MeOH); UV (MeOH) *λ*_max_ (log *ε*) 233 (4.7), 242 (4.1), 265 (5.0), 287 (2.2), 383 (2.0) nm; IR (KBr) *ν*_max_ 3449, 2920, 2362, 1651, 1579, 1428, 1274, 1040, 867 cm^–1^; NMR data, see Table 1 and Table 2; HRESIMS *m/z* 517.1826 [M + Na]^+^, (calcd. for C_28_H_30_O_8_Na, 517.1833).

Aspergixanthone K (**3**): yellow, amorphous powder; [α]${}_{20}^{D}$ = −94.0 (*c* 0.1, MeOH); UV (MeOH) *λ*_max_ (log *ε*) 232 (4.0), 243 (3.8), 267 (4.3), 286 (1.7), 384 (1.5) nm; IR (KBr) *ν*_max_ 3439, 2954, 2371, 1663, 1543, 1460, 1269, 1069, 935 cm^–1^; NMR data, see Table 1 and Table 2; HRESIMS *m/z* 453.1912 [M + Na]^+^, (calcd. for C_26_H_29_O_7_, 453.1908).

### 3.3. Preparation of the Methanolysis Derivative (***1a***) of ***1***

A solution of **1** (3.0 mg) and K_2_CO_3_ (10.0 mg) in anhydrous MeOH (3 mL) was stirred at room temperature for 5 h. The mixture was evaporated to dryness, and then purified using a silica gel column (PE/EtOAc, 1:1) to give the methanolysis derivative **1a** (2.5 mg).

Methanolysis derivative (**1a**): yellow, amorphous powder; ^1^H NMR (CDCl_3_, 600 MHz) *δ* 12.59 (1H, s, 1-OH), 7.52 (1H, d, *J* = 7.8 Hz, H-3), 7.23 (1H, s, H-5), 6.75 (1H, d, *J* = 7.8 Hz, H-2), 5.48 (1H, brs, H-25), 5.05 (1H, s, H-22a), 4.78 (1H, s, H-22b), 4.46 (1H, dd, *J* = 9.6, 1.8 Hz, H-19a), 4.32 (1H, dd, *J* = 10.2, 9.6 Hz, H-19b), 3.75 (1H, d, *J* = 9.6 Hz, H-15), 3.19 (1H, d, *J* = 13.8 Hz, H-14a), 2.68 (1H, dd, *J* = 13.8, 9.6 Hz, H-14b), 2.55 (1H, brd, *J* = 12.0 Hz, H-20), 2.36 (3H, s, H-24), 1.98 (3H, s, H-23), 1.43 (3H, s, H-18), and 1.35 (3H, s, H-17); ^13^C NMR (CDCl_3_, 150 MHz) *δ* 184.4 (C, C-13), 160.6 (C, C-1), 153.3 (C, C-10), 151.9 (C, C-11), 149.5 (C, C-7), 142.3 (C, C-21), 138.8 (C, C-6), 138.3 (CH, C-3), 121.7 (C, C-8), 119.4 (CH, C-5), 116.9 (C, C-12), 116.3 (C, C-4), 111.8 (CH_2_, C-22), 110.1 (CH, C-2), 109.4 (C, C-9), 77.9 (CH, C-15), 73.2 (C, C-16), 64.2 (CH_2_, C-19), 61.5 (CH, C-25), 44.1 (CH, C-20), 32.1 (CH_2_, C-14), 26.7 (CH_3_, C-18), 23.7 (CH_3_, C-17), 22.6 (CH_3_, C-23), and 17.6 (CH_3_, C-24); HRESIMS *m/z* 441.1906 [M + H]^+^, (calcd. for C_25_H_29_O_7_, 441.1908).

### 3.4. Snatzke’s Method

The ICD spectra of **1a** and **4** were obtained after addition of Mo_2_(OAc)_4_ following a previously referenced procedure [16,17].

### 3.5. Anti-Vibrio Activity Assays

Anti-*Vibrio* activity was evaluated by the conventional broth dilution assay [20]. Three pathogenic *Vibrio* strains, *Vibrio parahemolyticus, Vibrio anguillarum*, and *Vibrio* *alginolyticus* were used, and ciprofloxacin was used as a positive control with MIC values of 0.078 μM, 0.312 μM, and 0.625 μM, respectively. Replicates were maintained for each test bacteria.

## 4. Conclusions

Seven prenylxanthone derivatives, including three new compounds (**1**–**3**), were obtained from the marine-derived fungus *Aspergillus* sp. ZA-01 by using a shaken Czapek-Dox medium. The absolute configuration of **1** was determined by the Mo_2_(AcO)_4_ ICD method. This work suggested that the OSMAC approach was an active pathway for the exploration of new bioactive molecules.

## Figures and Tables

**Table 1:** ^1^H NMR data (*δ*) of **1**–**3** (600 MHz, *δ* in ppm, CDCl_3_, *J* in Hz).

Position	1	2	3
2	6.71, d (8.4)	6.80, d (8.4)	6.85, d (8.4)
3	7.41, d (8.4)	7.60, d (8.4)	7.65, d (8.4)
5	7.29, s	7.27, s	7.24, s
14	3.33, dd (14.4, 2.4)	4.82, d (8.4)	4.83, d (8.4)
	2.91, dd (14.4, 10.8)		
15	5.15, dd (10.8, 2.4)	4.19, d (8.4)	4.19, d (8.4)
17	1.34, s	4.65, brs	4.64, brs
		4.62, brs	4.60, brs
18	1.38, s	1.76, s	1.77, s
19	4.46, brd (10.8)	4.56, brd (11.4)	4.43, dd (10.8, 3.0)
	4.35, dd (12.0, 10.8)	4.32, dd (11.4, 3.0)	4.35, dd (10.8, 3.0)
20	2.55, d (12.0)	2.72, brs	2.72, d (3.0)
22	5.06, s	4.81, s	4.81, s
	4.78, s	4.76, s	4.59, s
23	1.86, s	1.89, s	1.85, s
24	2.38, s	2.36, s	2.37, s
25	5.50, brs	6.90, brs	5.43, brs
1-OH	12.63, brs	13.06, brs	12.83, brs
14-OCH_3_	-	3.28, s	3.30, s
15-OAc	1.99, s	-	-
25-OH	4.51, brs	-	4.96, d (4.2)
25-OAc	-	2.10, s	-

**Table 2:** ^13^C NMR data (*δ*) of **1**–**3** (150 MHz, *δ* in ppm, CDCl_3_).

Position	1	2	3
1	161.1, C	162.0, C	161.8, C
2	109.5, CH	110.7, CH	110.7, CH
3	137.9, CH	134.7, CH	135.1, CH
4	115.1, C	115.4, C	115.8, C
5	119.5, CH	120.4, CH	119.1, CH
6	139.0, C	138.0, C	139.0, C
7	149.6, C	150.4, C	149.9, C
8	121.8, C	115.0, C	121.4, C
9	109.2, C	109.0, C	108.8, C
10	153.3, C	153.5, C	153.7, C
11	151.8, C	151.8, C	152.0, C
12	116.9, C	116.4, C	116.9, C
13	184.5, C	183.4, C	184.5, C
14	29.7, CH_2_	78.7, CH	78.8, CH
15	78.6, CH	80.0, CH	80.0, CH
16	72.5, C	141.7, C	142.5, C
17	26.9, CH_3_	114.8, CH_2_	114.8, CH_2_
18	25.3, CH_3_	18.2, CH_3_	18.2, CH_3_
19	64.1, CH_2_	63.9, CH_2_	64.8, CH_2_
20	44.1, CH	42.6, CH	45.1, CH
21	142.3, C	142.5, C	142.7, C
22	111.7, CH_2_	112.9, CH_2_	112.4, CH_2_
23	22.5, CH_3_	22.6, CH_3_	22.7, CH_3_
24	17.4, CH_3_	17.5, CH_3_	17.7, CH_3_
25	61.0, CH	65.7, CH	63.3, CH
14-OCH_3_	-	57.2, CH_3_	57.2, CH_3_
15-OAc	170.4, C	-	-
	20.7, CH_3_		
25-OAc	-	170.2, C	-
		21.4, CH_3_	

**Table 3:** Tests of anti-*Vibrio* activities for compounds **1**–**7**.

Strains	Compounds [MIC (μM)]							
	1	2	3	4	5	6	7	Ciprofloxacin
*V. parahemolyticus*	1.56	6.25	3.12	25.0	12.5	6.25	25.0	0.078
*V. anguillarum*	1.56	25.0	25.0	25.0	25.0	6.25	6.25	0.312
*V. alginolyticus*	3.12	25.0	12.5	25.0	12.5	12.5	25.0	0.625

## Supplementary Materials

The following are available online at http://www.mdpi.com/1660-3397/16/9/312/s1. Figures S1–S24: 1D and 2D NMR, and mass spectra of **1**–**3**.
