# Supplementary material for: Aspergixanthones I–K, New Anti-Vibrio Prenylxanthones from the Marine-Derived Fungus Aspergillus sp. ZA-01
Source: Mar Drugs. 2018 Sep 4;16(9):312. doi: 10.3390/md16090312 (PMC6165128; doi:10.3390/md16090312)
Supplement: Supplementary file 1 [file marinedrugs-16-00312-s001.pdf]

List of Supporting Information

Figure S1. ^{1}H NMR (600 MHz, $CDCl_3$) spectrum of compound **1**

Figure S2. ^{13}C NMR (150 MHz, $CDCl_3$) spectrum of compound **1**

Figure S3. HSQC ($CDCl_3$) spectrum of compound **1**

Figure S4. ^{1}H-^{1}H COSY ($CDCl_3$) spectrum of compound **1**

Figure S5. HMBC ($CDCl_3$) spectrum of compound **1**

Figure S6. NOESY ($CDCl_3$) spectrum of compound **1**

Figure S7. HRESIMS spectrum of compound **1**

Figure S8. ^{1}H NMR (600 MHz, $CDCl_3$) spectrum of compound **1a**

Figure S9. ^{13}C NMR (150 MHz, $CDCl_3$) spectrum of compound **1a**

Figure S10. HRESIMS spectrum of compound **1a**

Figure S11. ^{1}H NMR (600 MHz, $CDCl_3$) spectrum of compound **2**

Figure S12. ^{13}C NMR (150 MHz, $CDCl_3$) spectrum of compound **2**

Figure S13. HSQC ($CDCl_3$) spectrum of compound **2**

Figure S14. ^{1}H-^{1}H COSY ($CDCl_3$) spectrum of compound **2**

Figure S15. HMBC ($CDCl_3$) spectrum of compound **2**

Figure S16. NOESY ($CDCl_3$) spectrum of compound **2**

Figure S17. HRESIMS spectrum of compound **2**

Figure S18. ^{1}H NMR (600 MHz, $CDCl_3$) spectrum of compound **3**

Figure S19. ^{13}C NMR (150 MHz, $CDCl_3$) spectrum of compound **3**

Figure S20. HSQC ($CDCl_3$) spectrum of compound **3**

Figure S21. ^{1}H-^{1}H COSY ($CDCl_3$) spectrum of compound **3**

Figure S22. HMBC ($CDCl_3$) spectrum of compound **3**

Figure S23. NOESY ($CDCl_3$) spectrum of compound **3**

Figure S24. HRESIMS spectrum of compound **3**

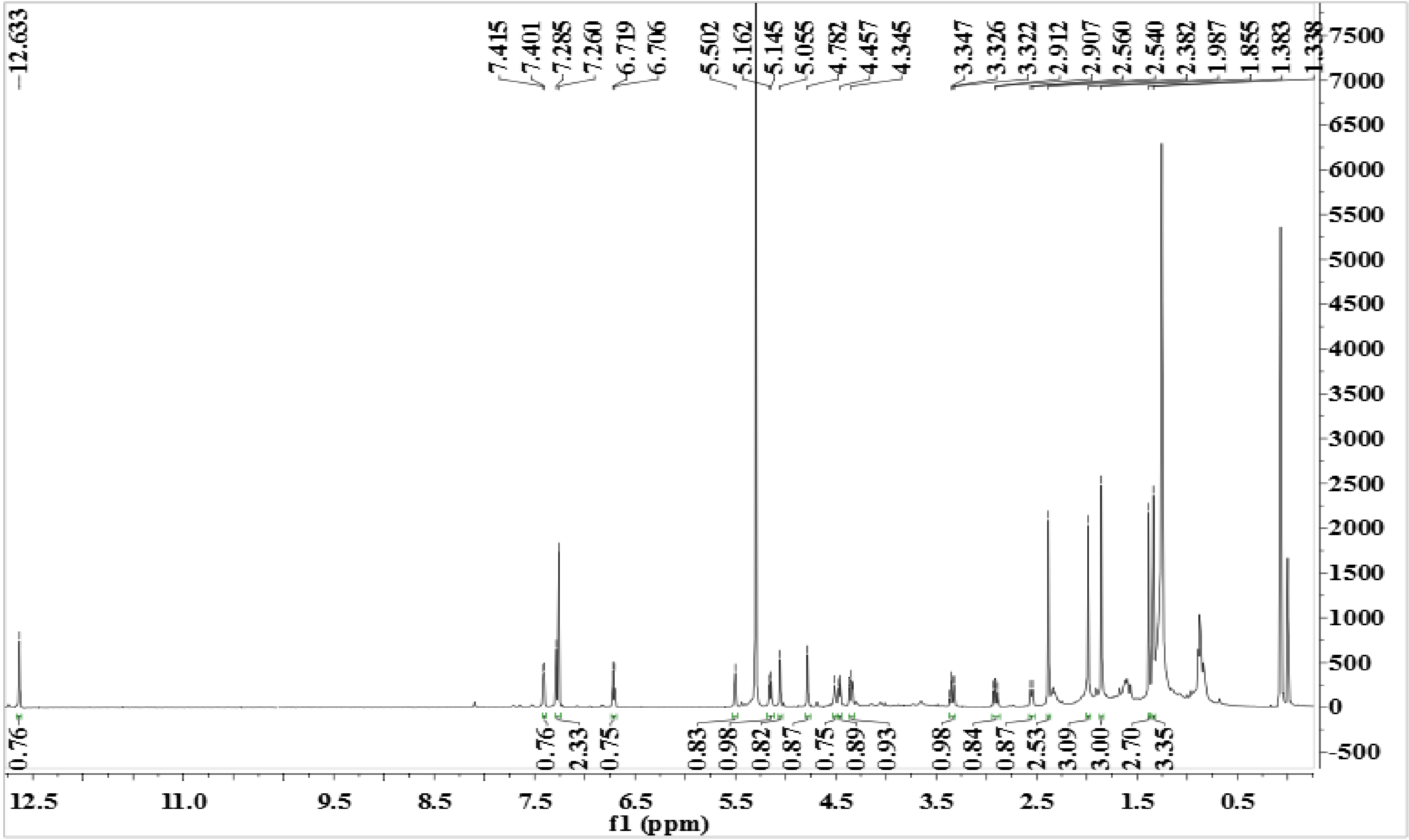

Figure S1. ^1H NMR (600 MHz, $CDCl_3$) spectrum of compound **1**

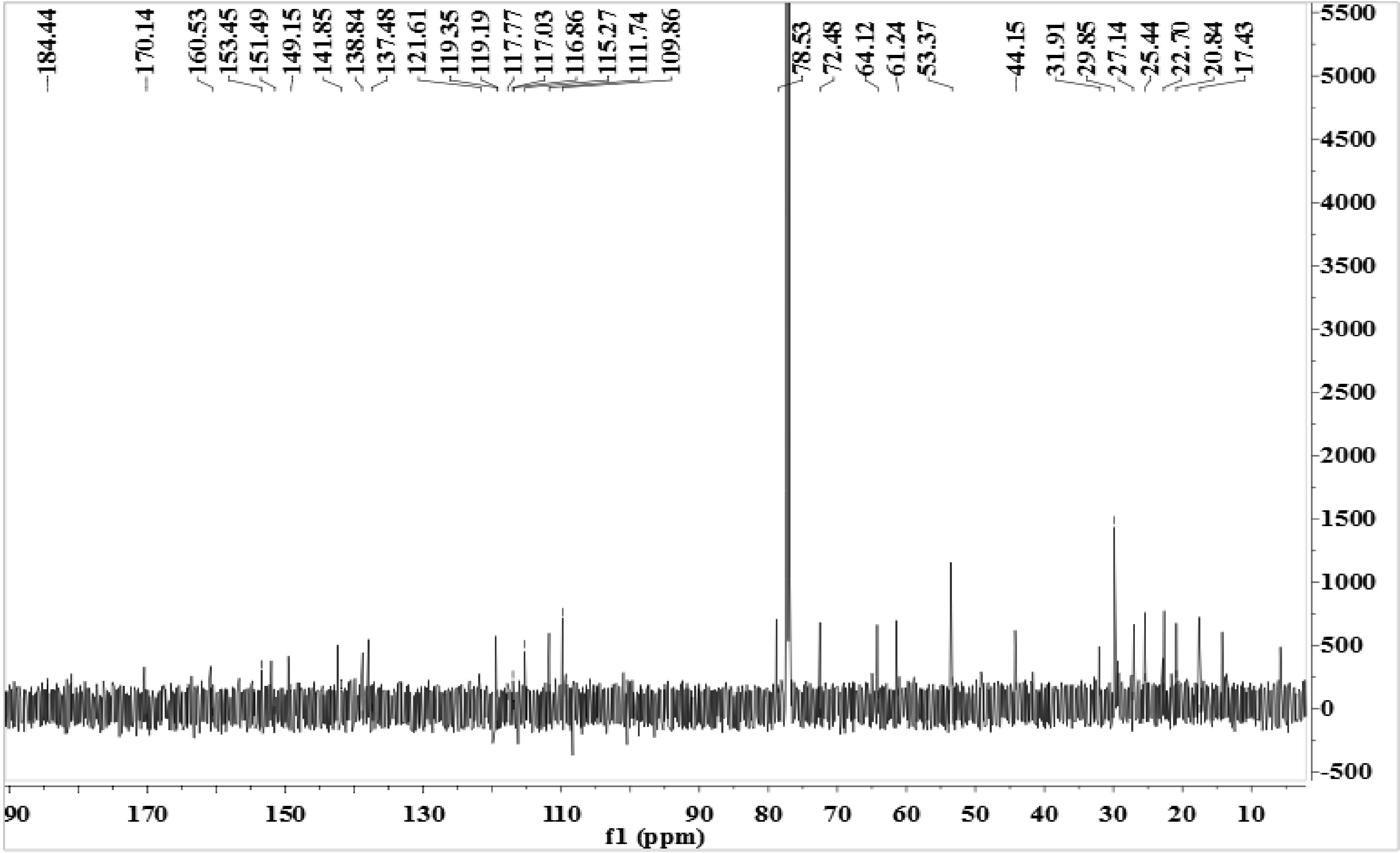

Figure S2. ^{13}C NMR (150 MHz, $CDCl_3$) spectrum of compound **1**

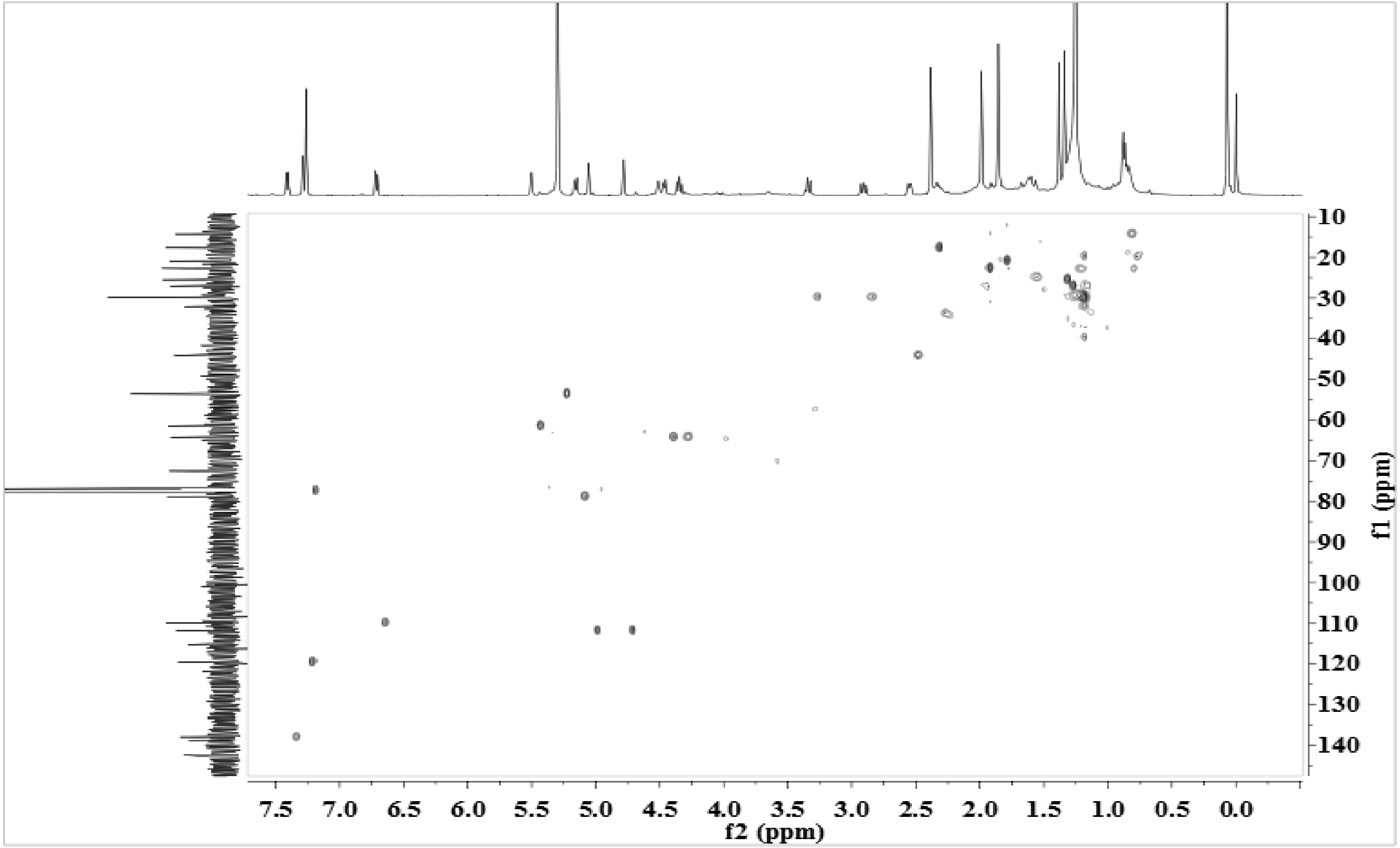

Figure S3. HSQC ($CDCl_3$) spectrum of compound **1**

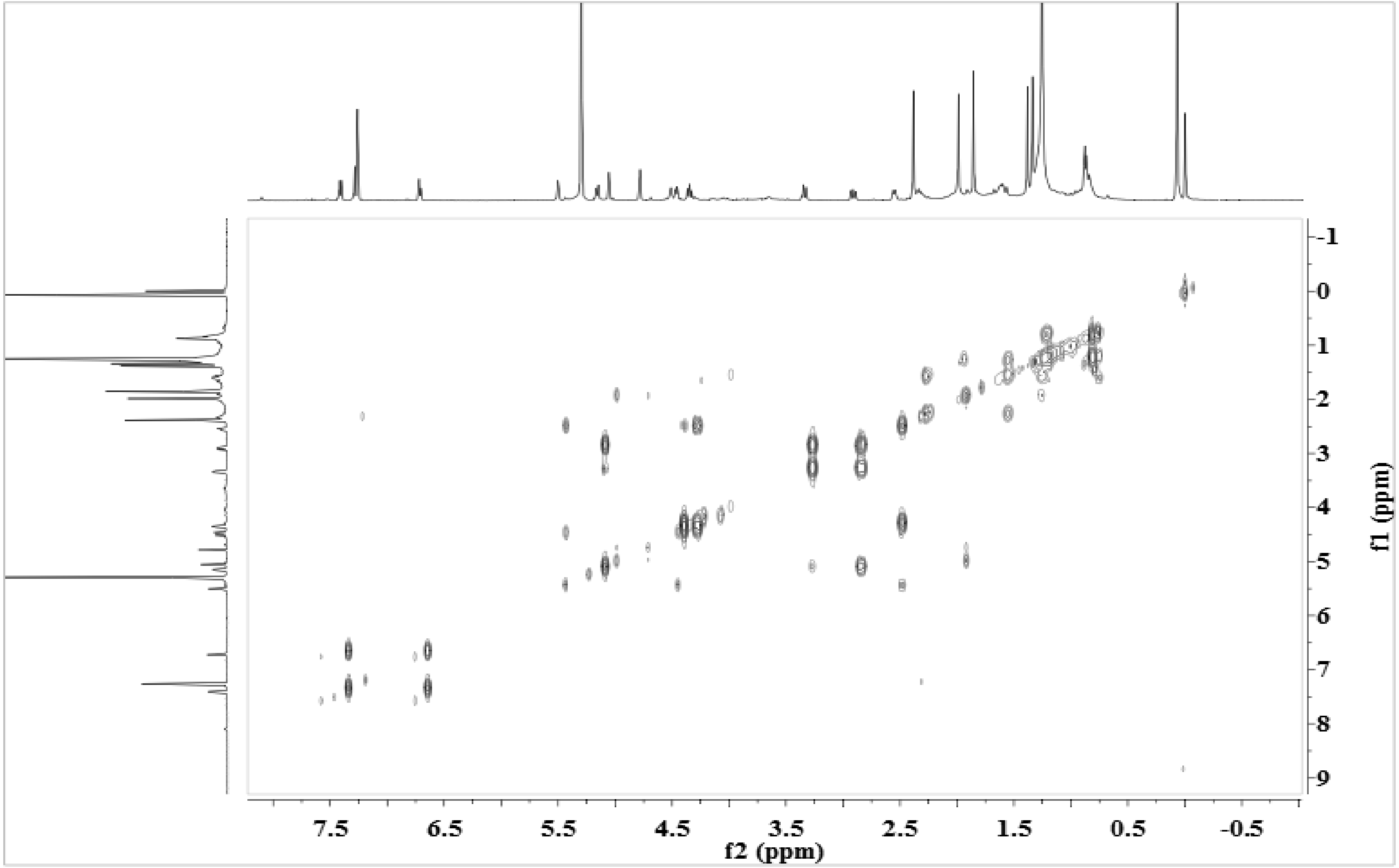

Figure S4. ^{1}H-^{1}H COSY ($CDCl_3$) spectrum of compound **1**

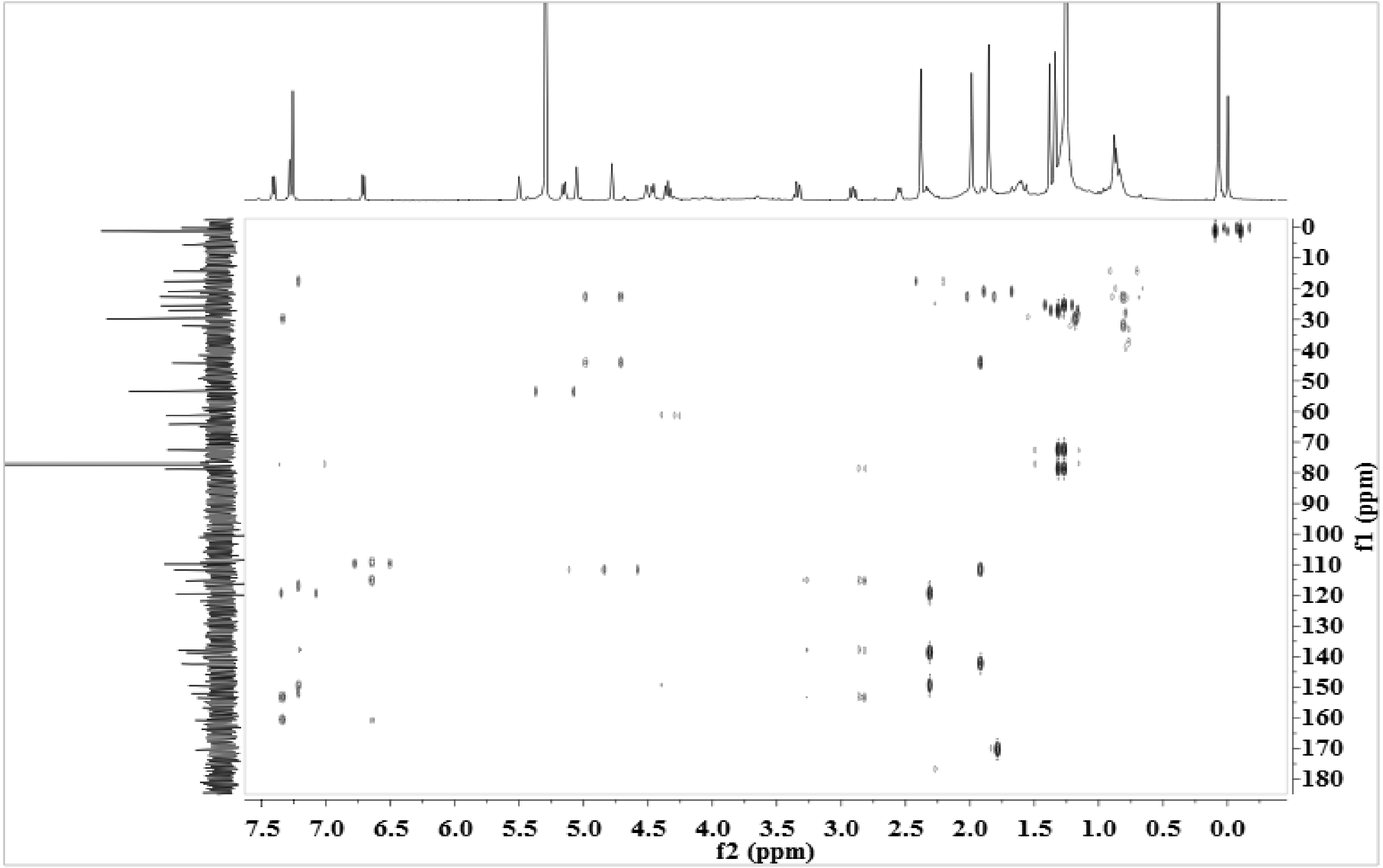

Figure S5. HMBC ($CDCl_3$) spectrum of compound **1**

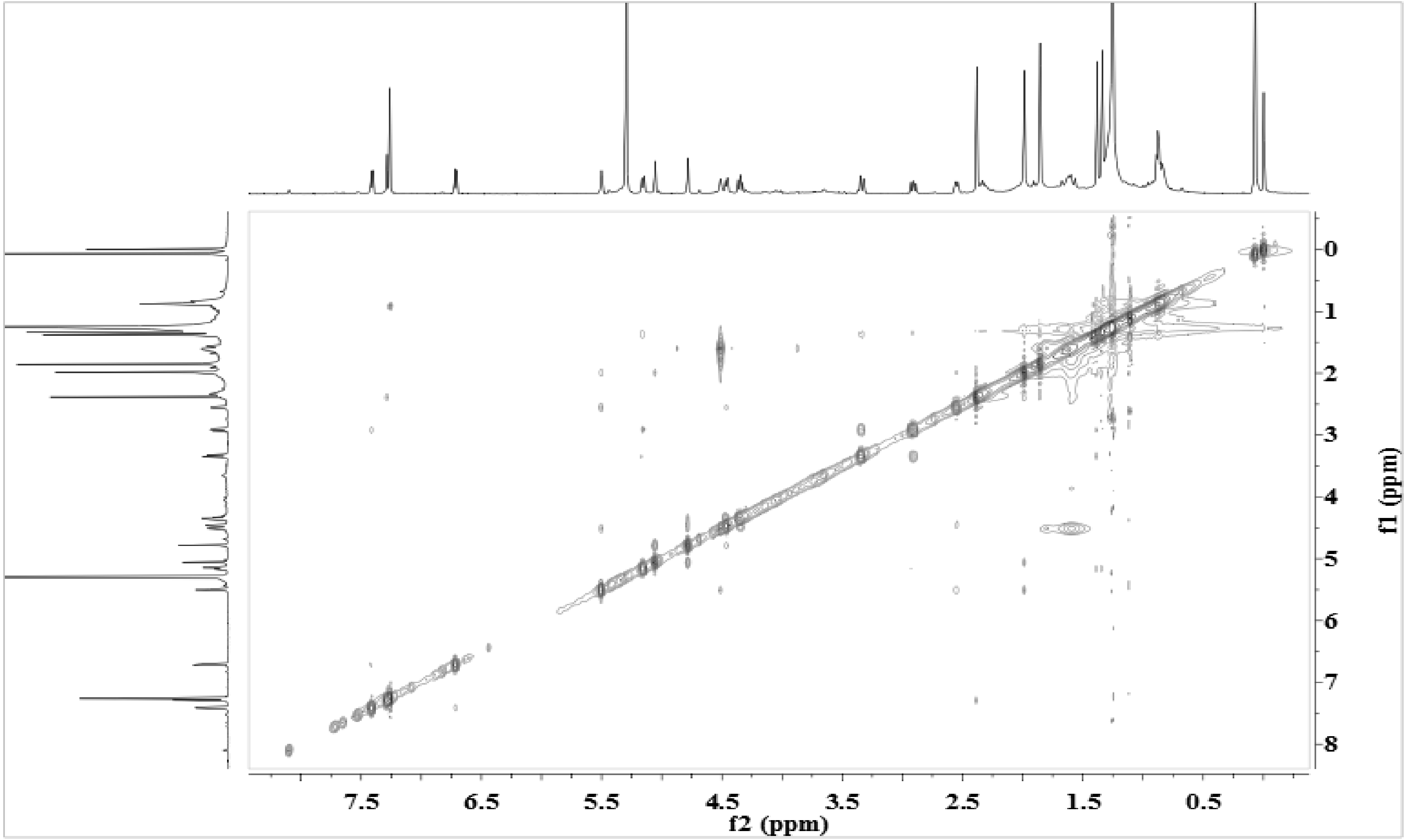

Figure S6. NOESY ($CDCl_3$) spectrum of compound **1**

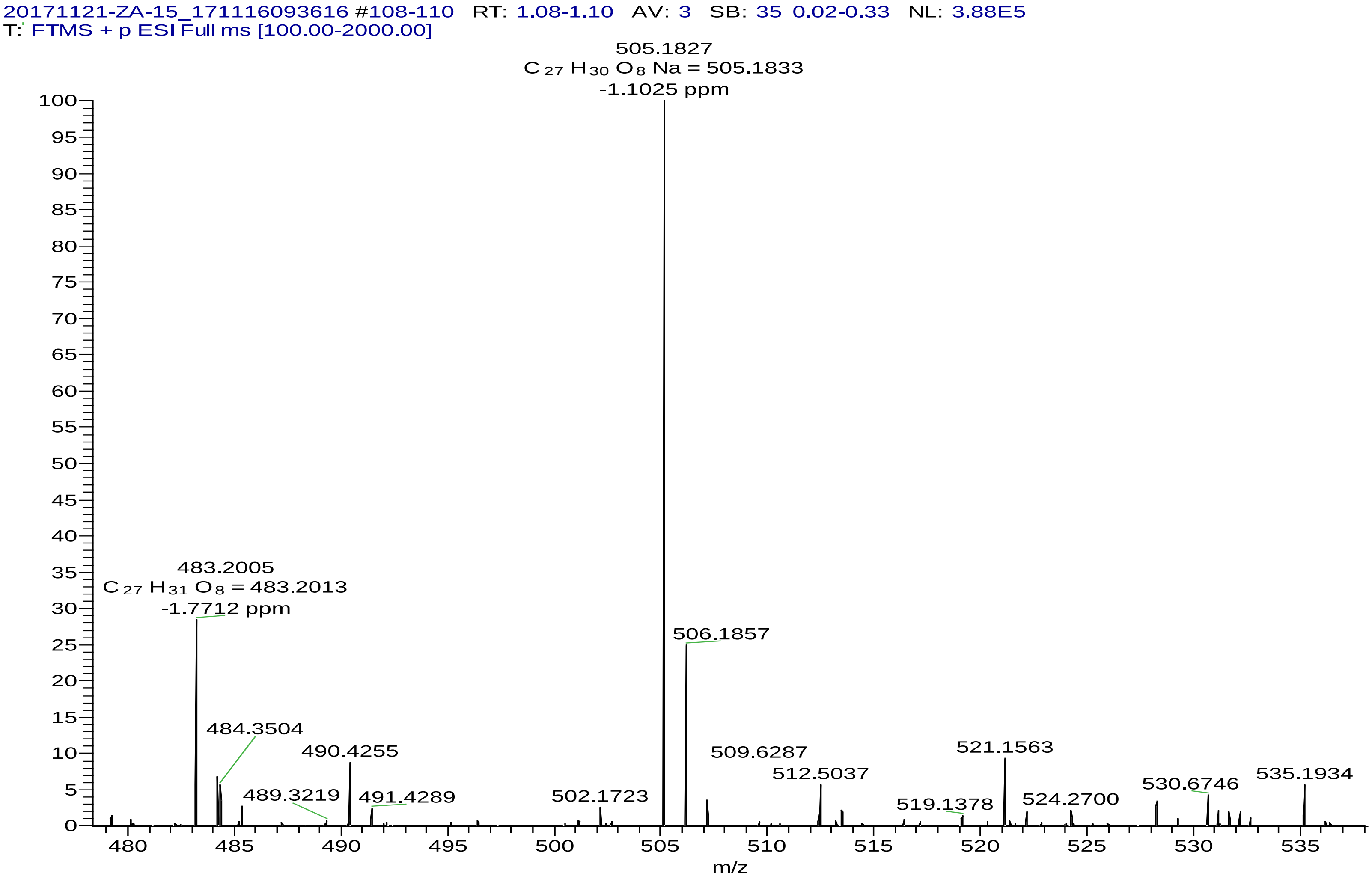

Figure S7. HRESIMS spectrum of compound **1**

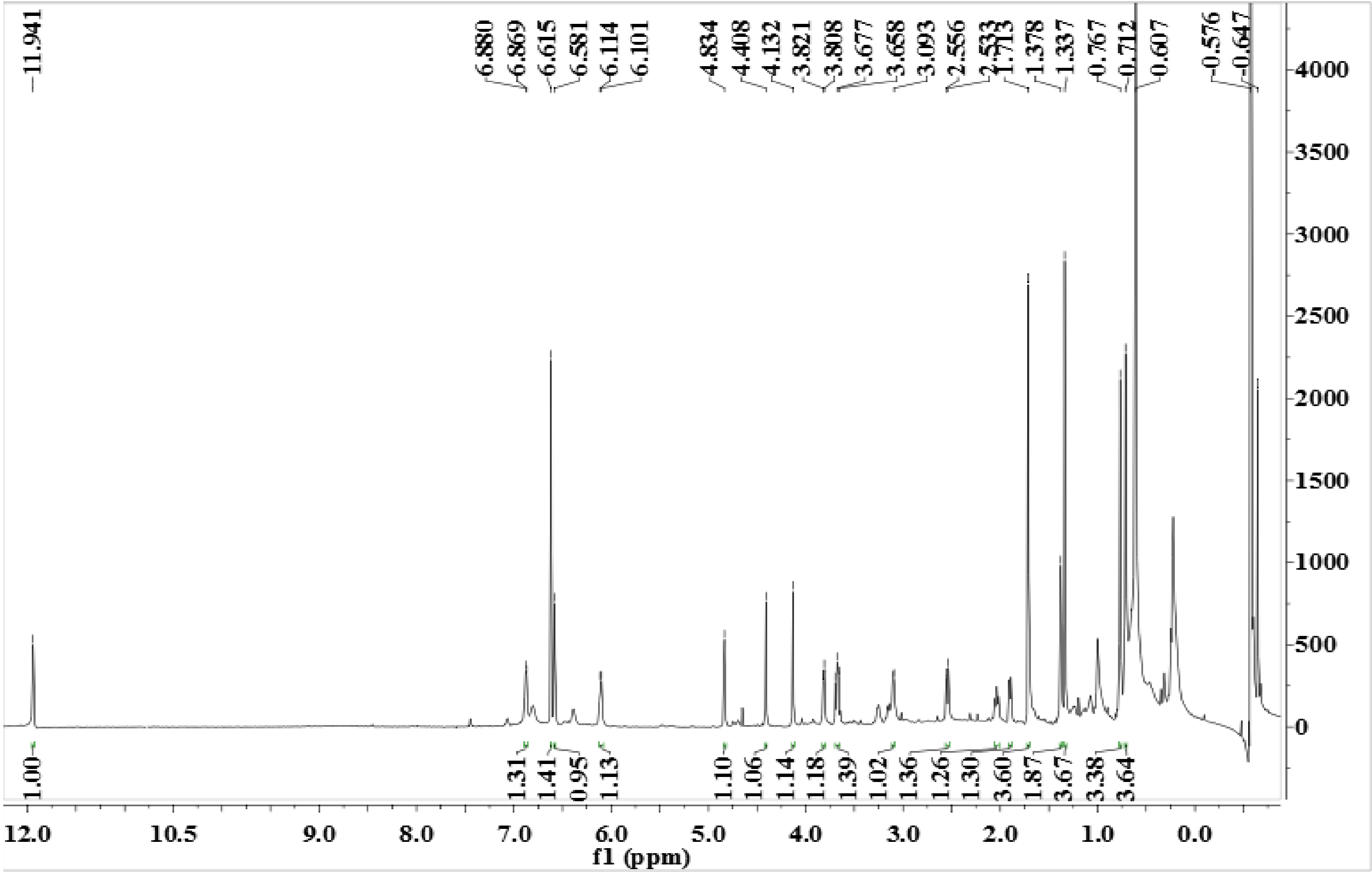

Figure S8. ^{1}H NMR (600 MHz, $CDCl_3$) spectrum of compound **1a**

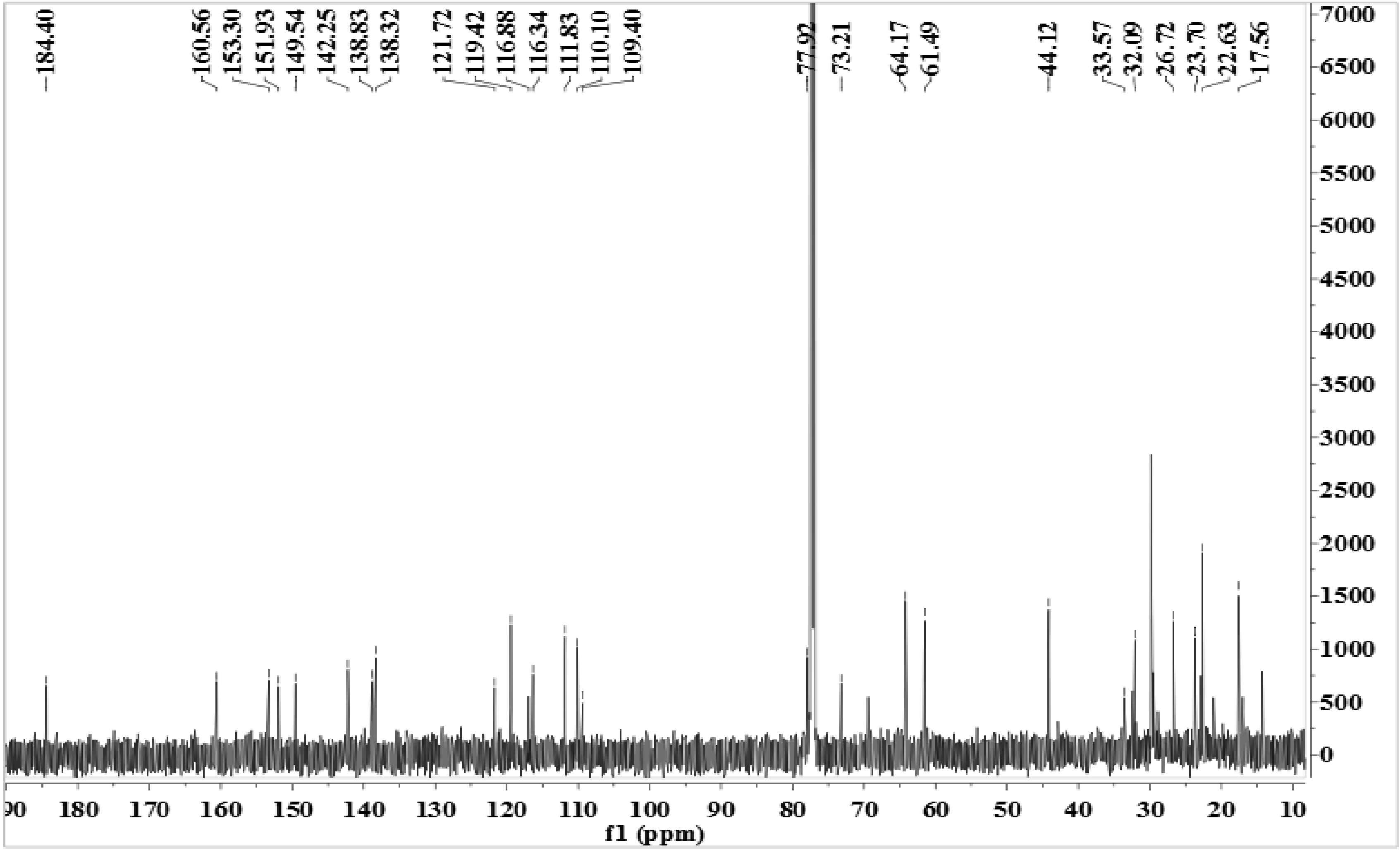

Figure S9. ^{13}C NMR (150 MHz, $CDCl_3$) spectrum of compound **1a**

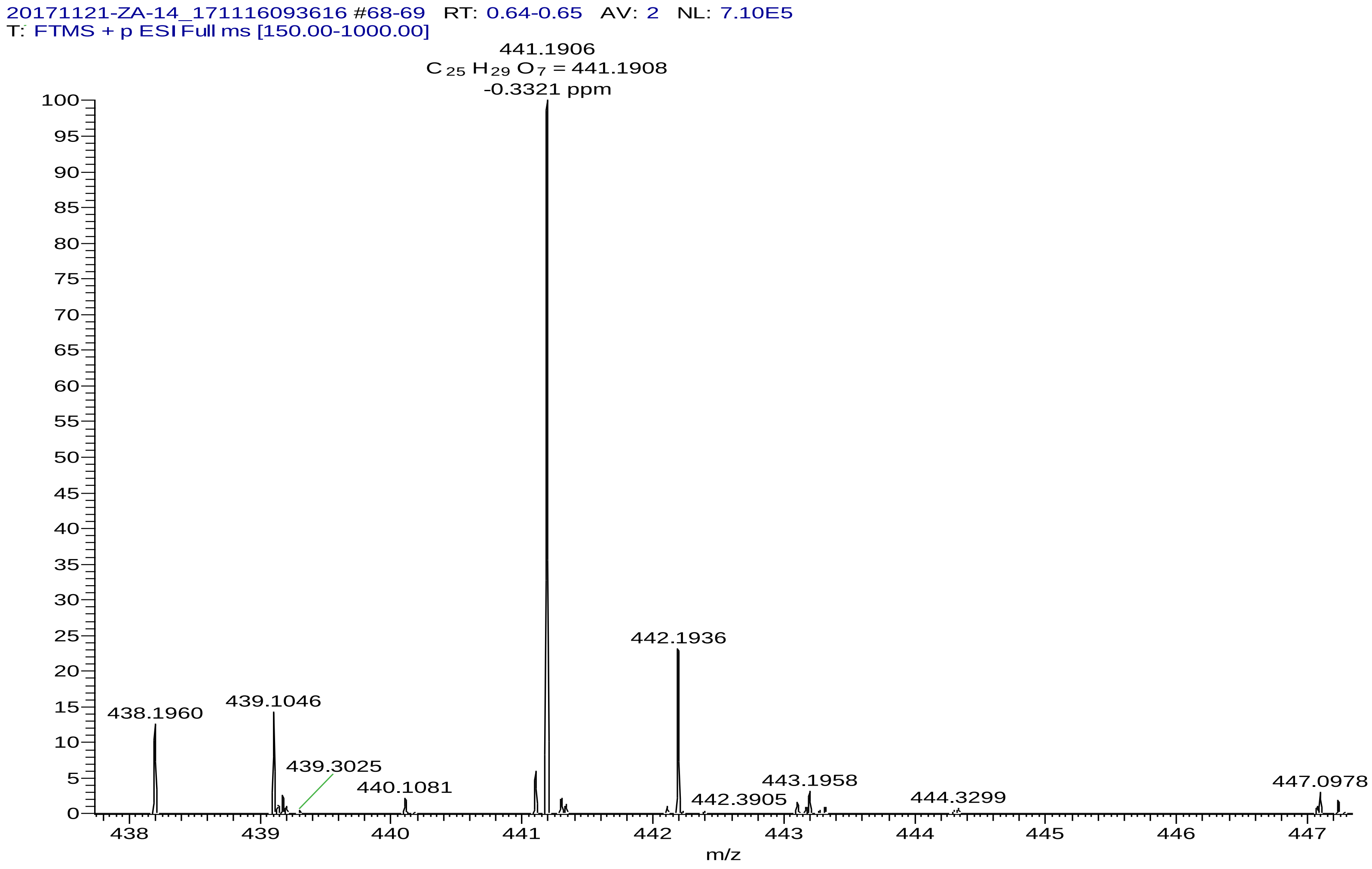

Figure S10. HRESIMS spectrum of compound **1a**

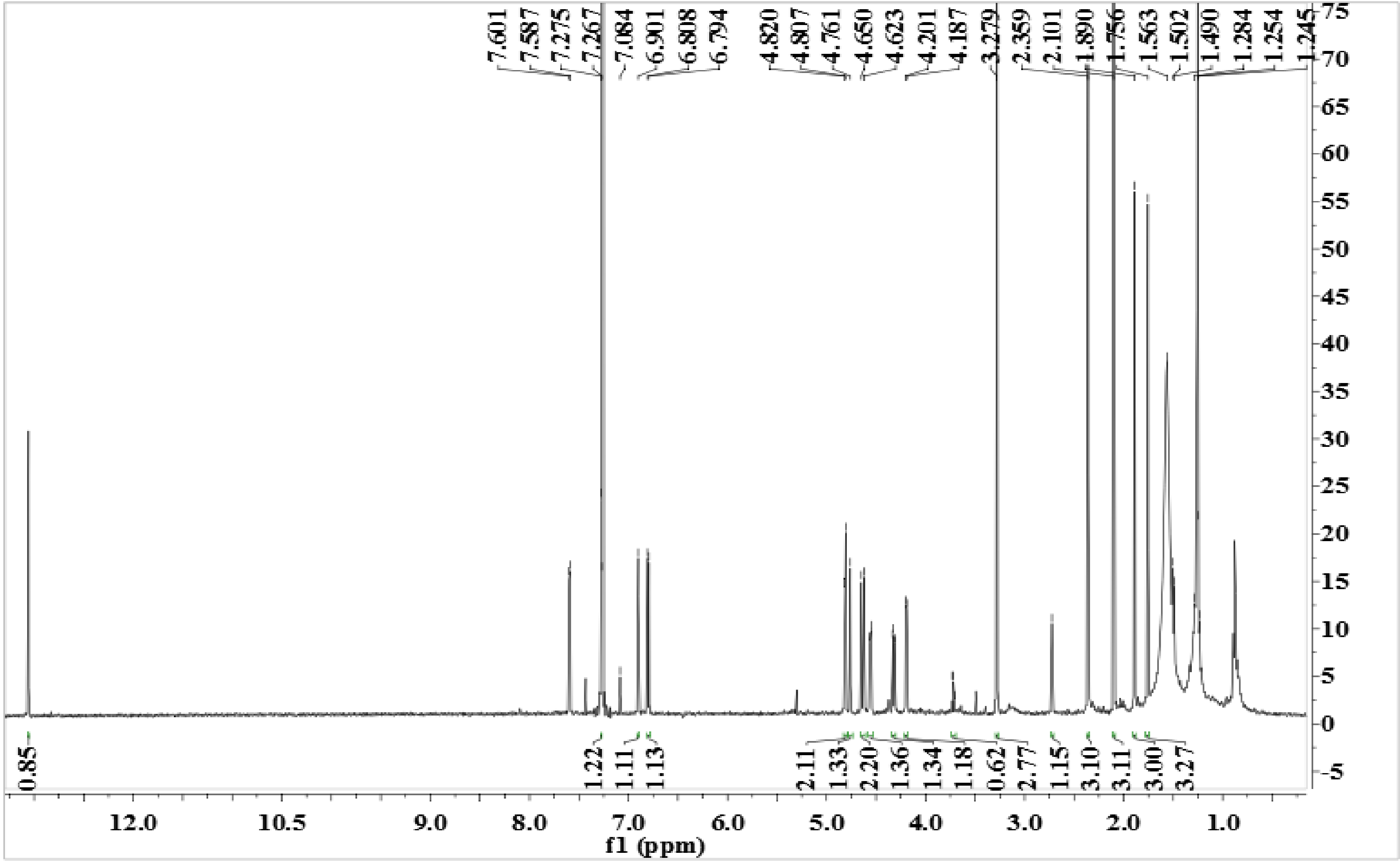

Figure S11. ^{1}H NMR (600 MHz, $CDCl_3$) spectrum of compound **2**

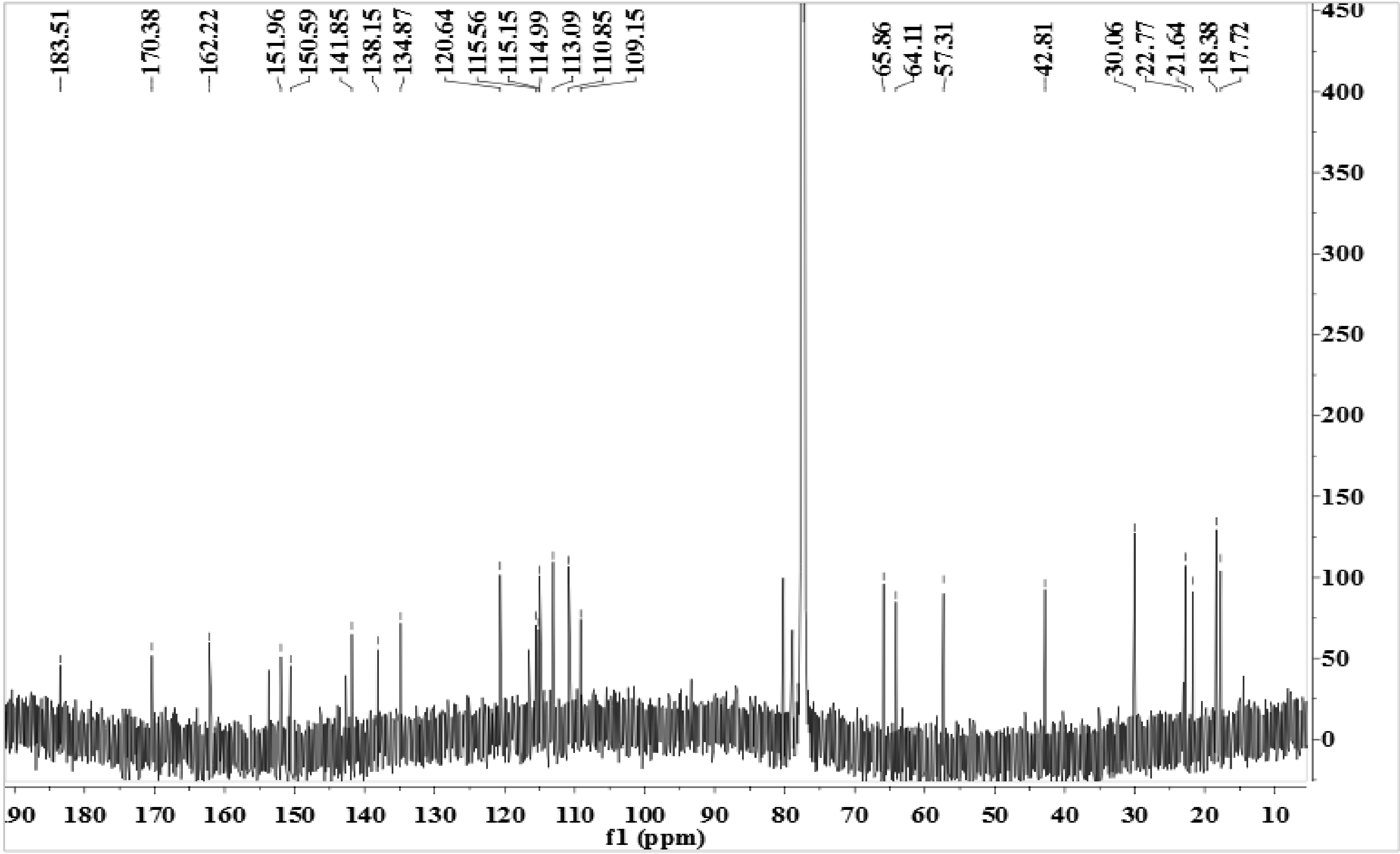

Figure S12. ^{13}C NMR (150 MHz, $CDCl_3$) spectrum of compound **2**

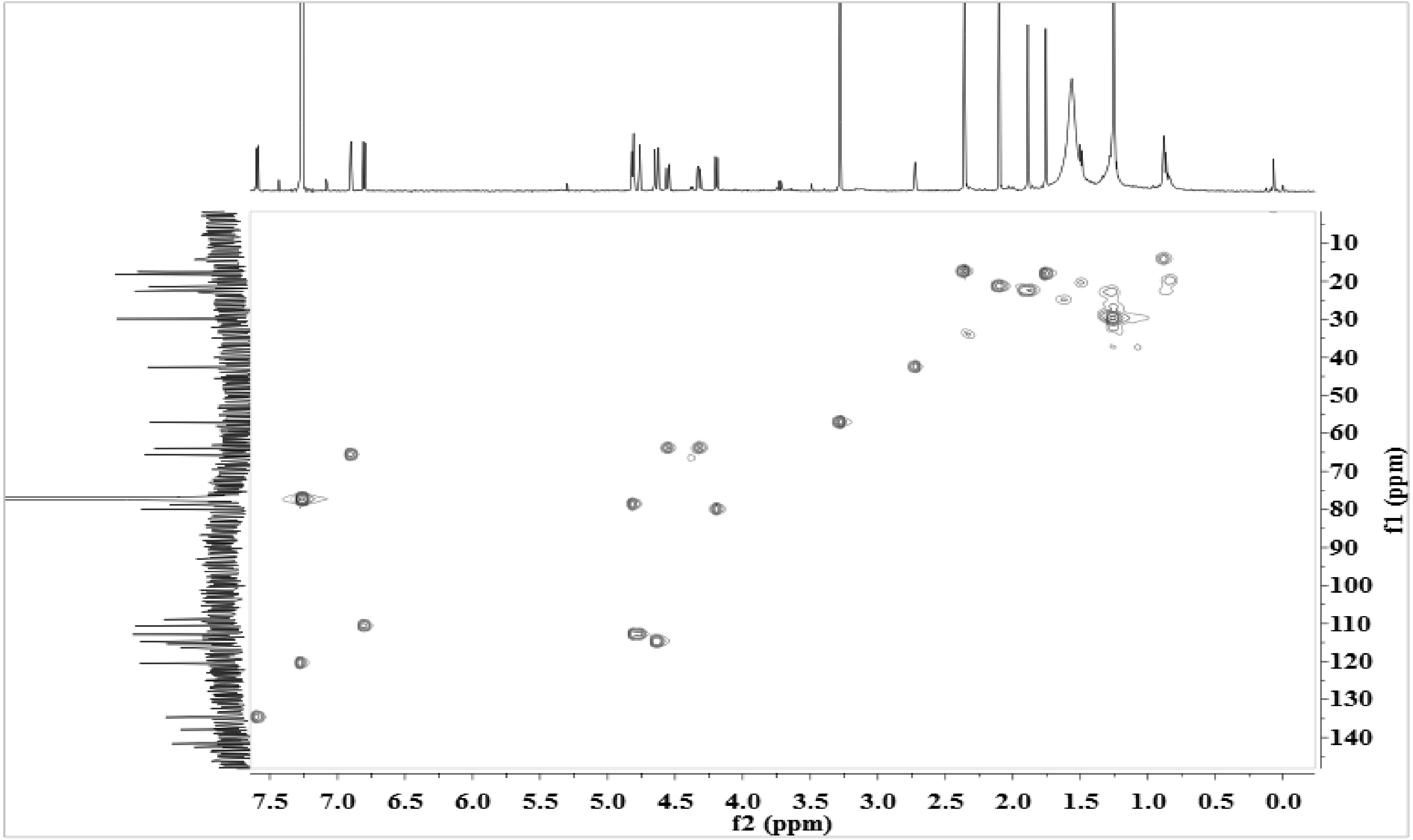

Figure S13. HSQC ($CDCl_3$) spectrum of compound **2**

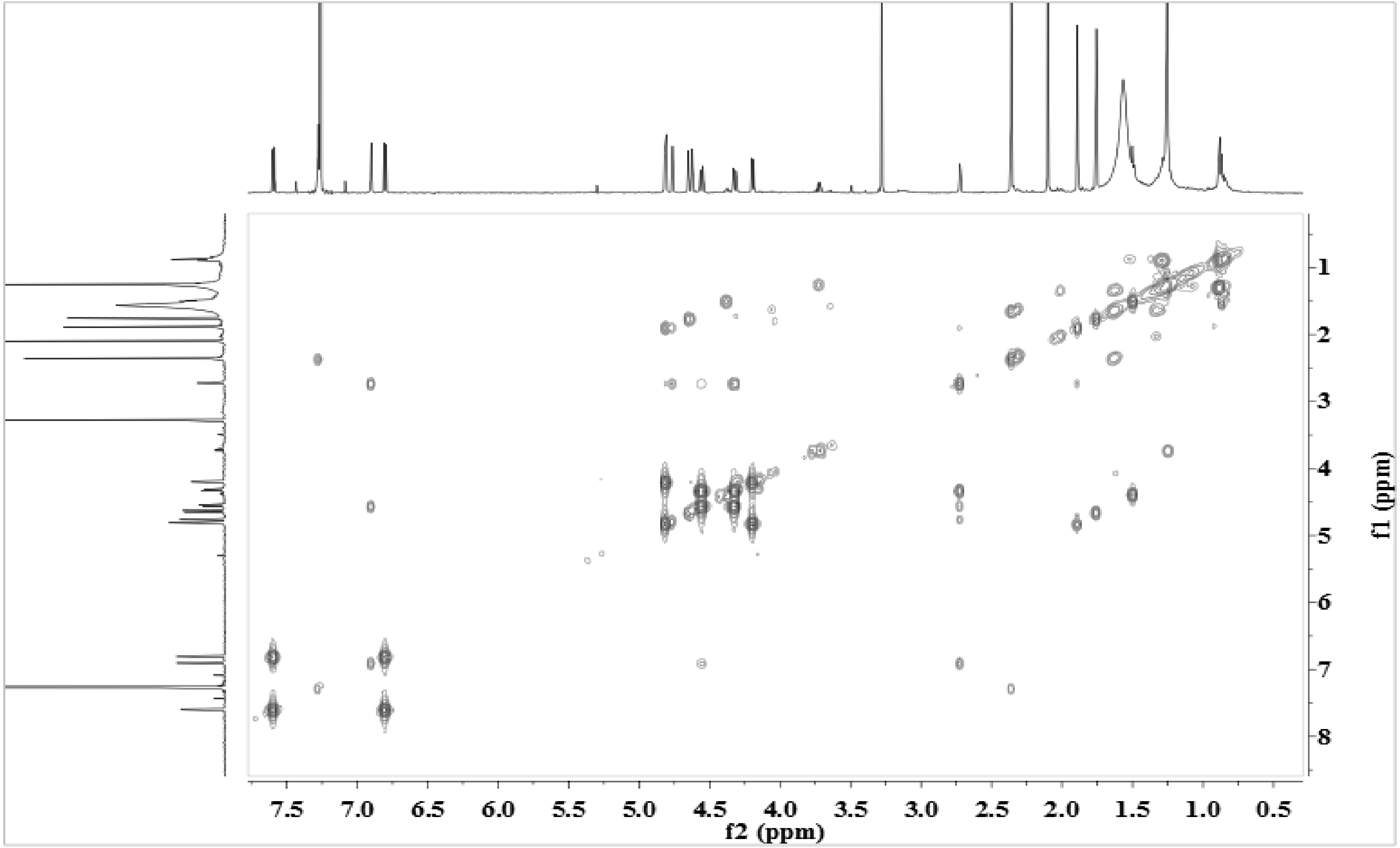

Figure S14. ^{1}H-^{1}H COSY ($CDCl_3$) spectrum of compound **2**

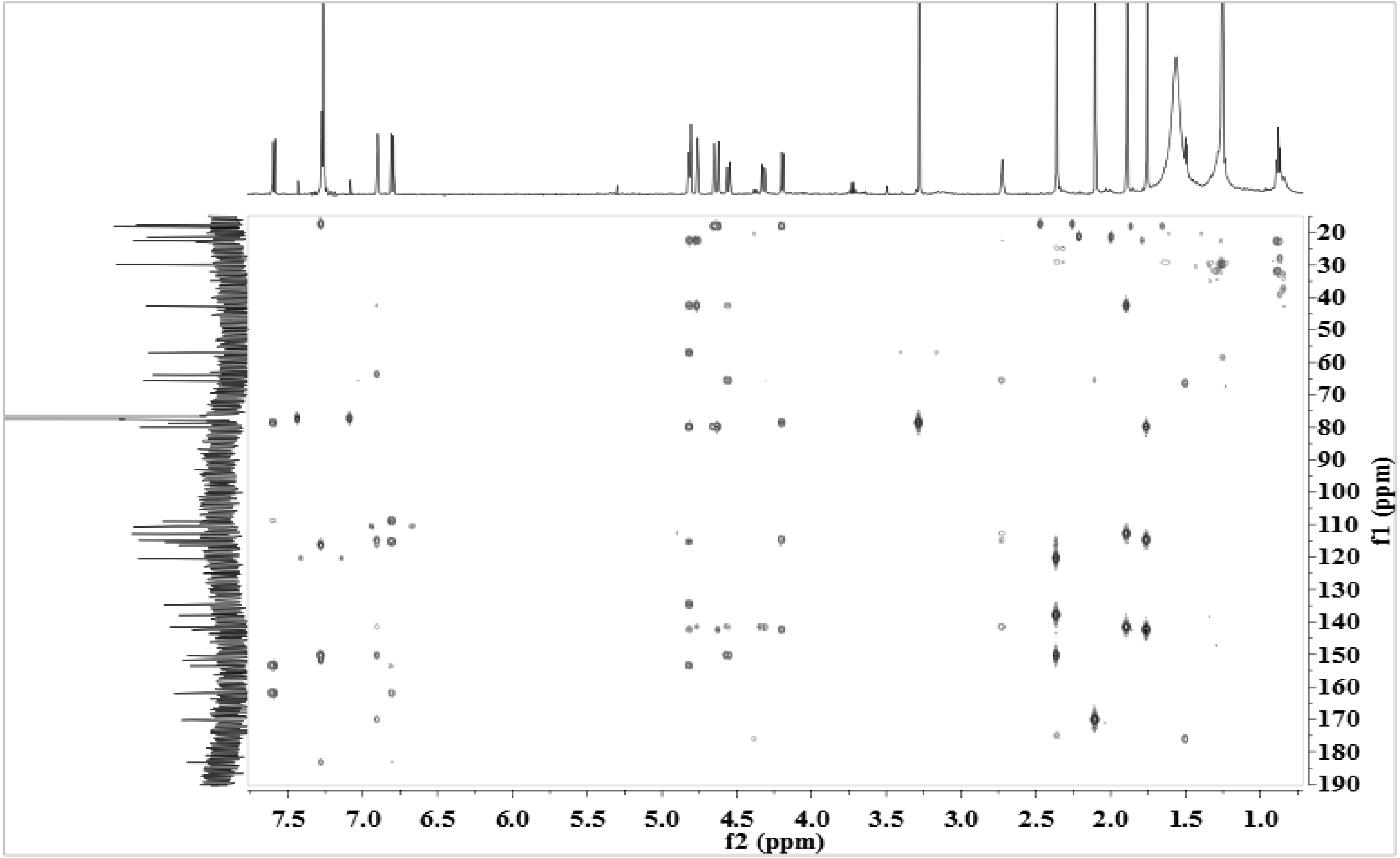

Figure S15. HMBC ($CDCl_3$) spectrum of compound **2**

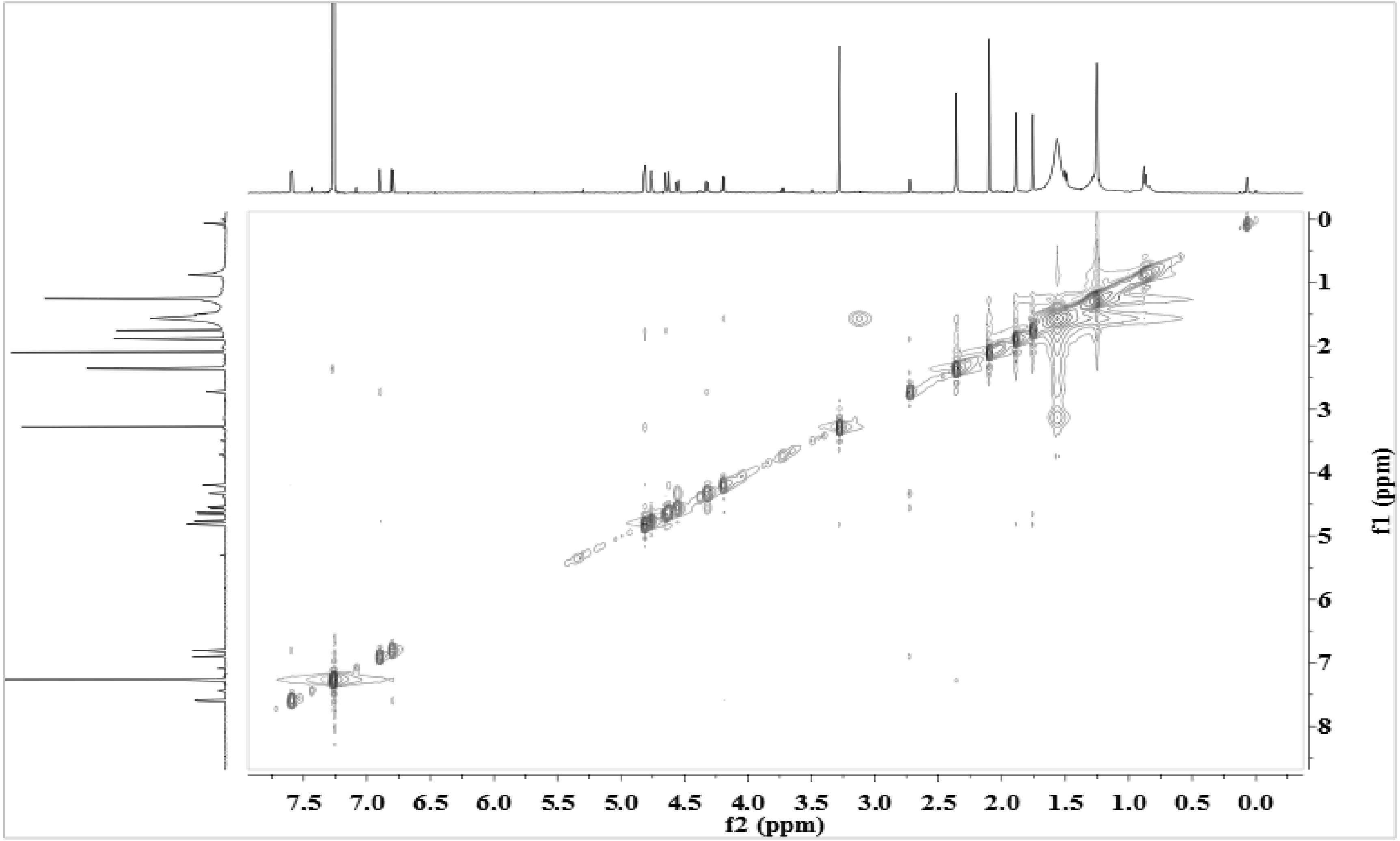

Figure S16. NOESY ($CDCl_3$) spectrum of compound **2**

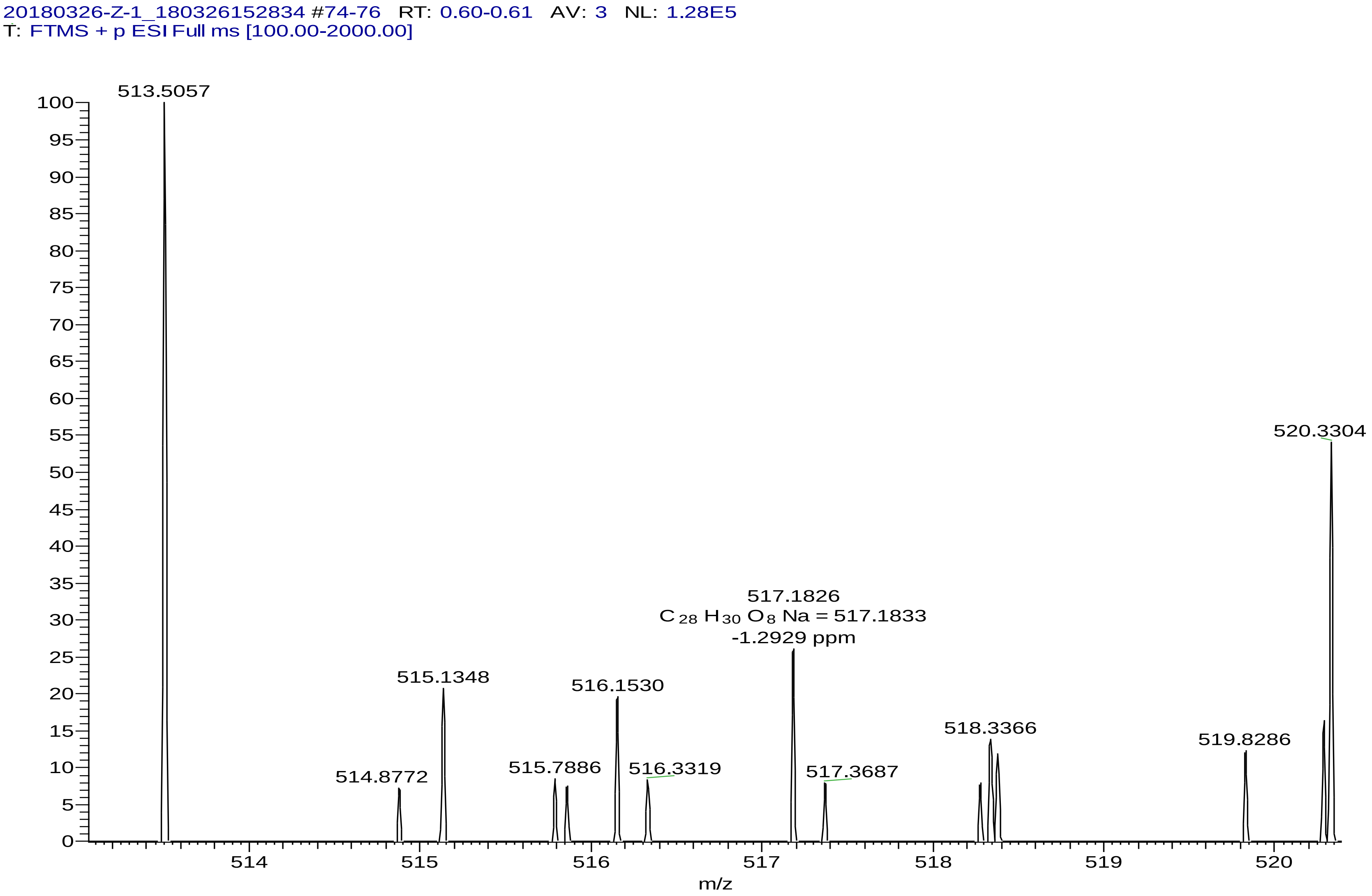

Figure S17. HRESIMS spectrum of compound **2**

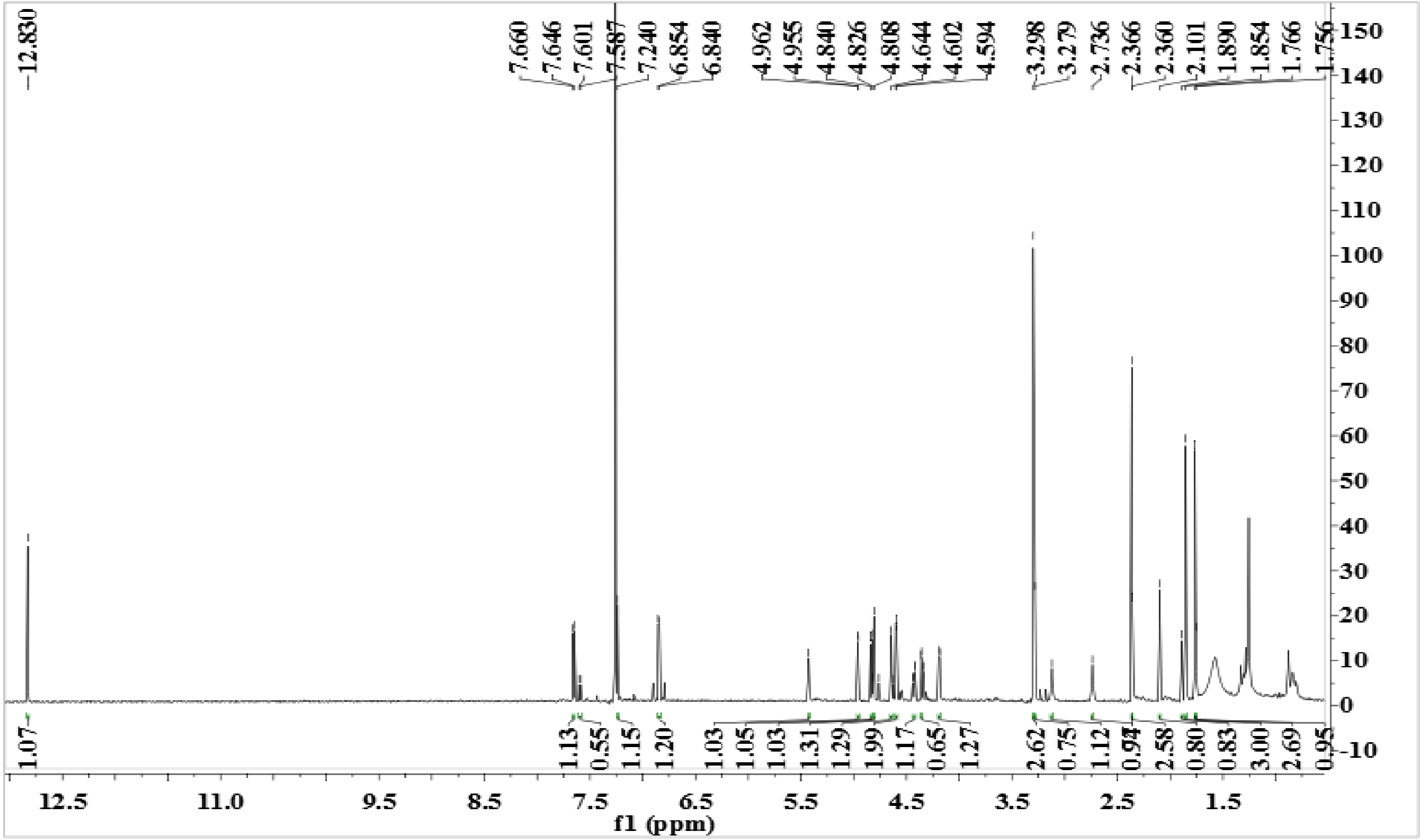

Figure S18. ^{1}H NMR (600 MHz, $CDCl_3$) spectrum of compound **3**

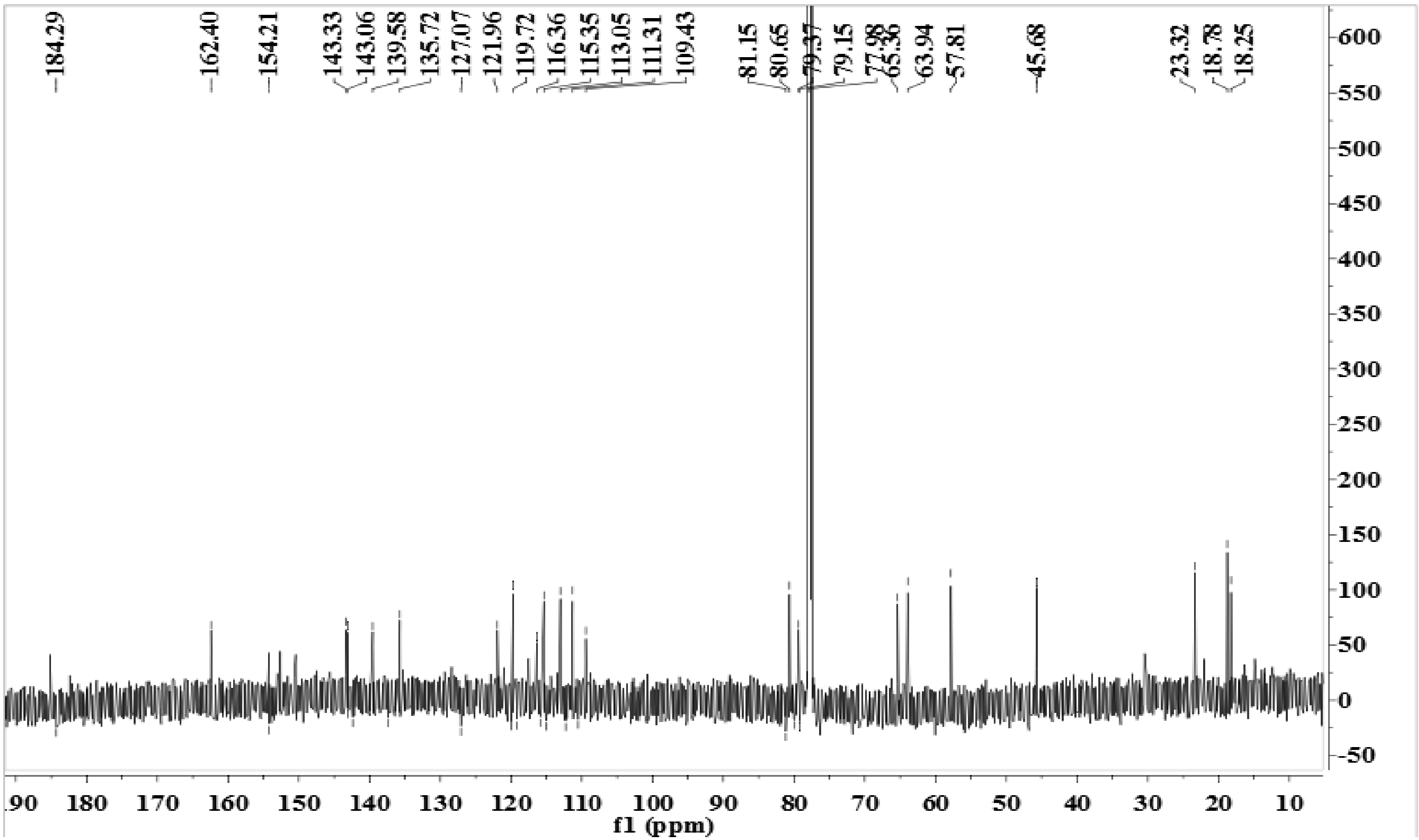

Figure S19. ^{13}C NMR (150 MHz, $CDCl_3$) spectrum of compound **3**

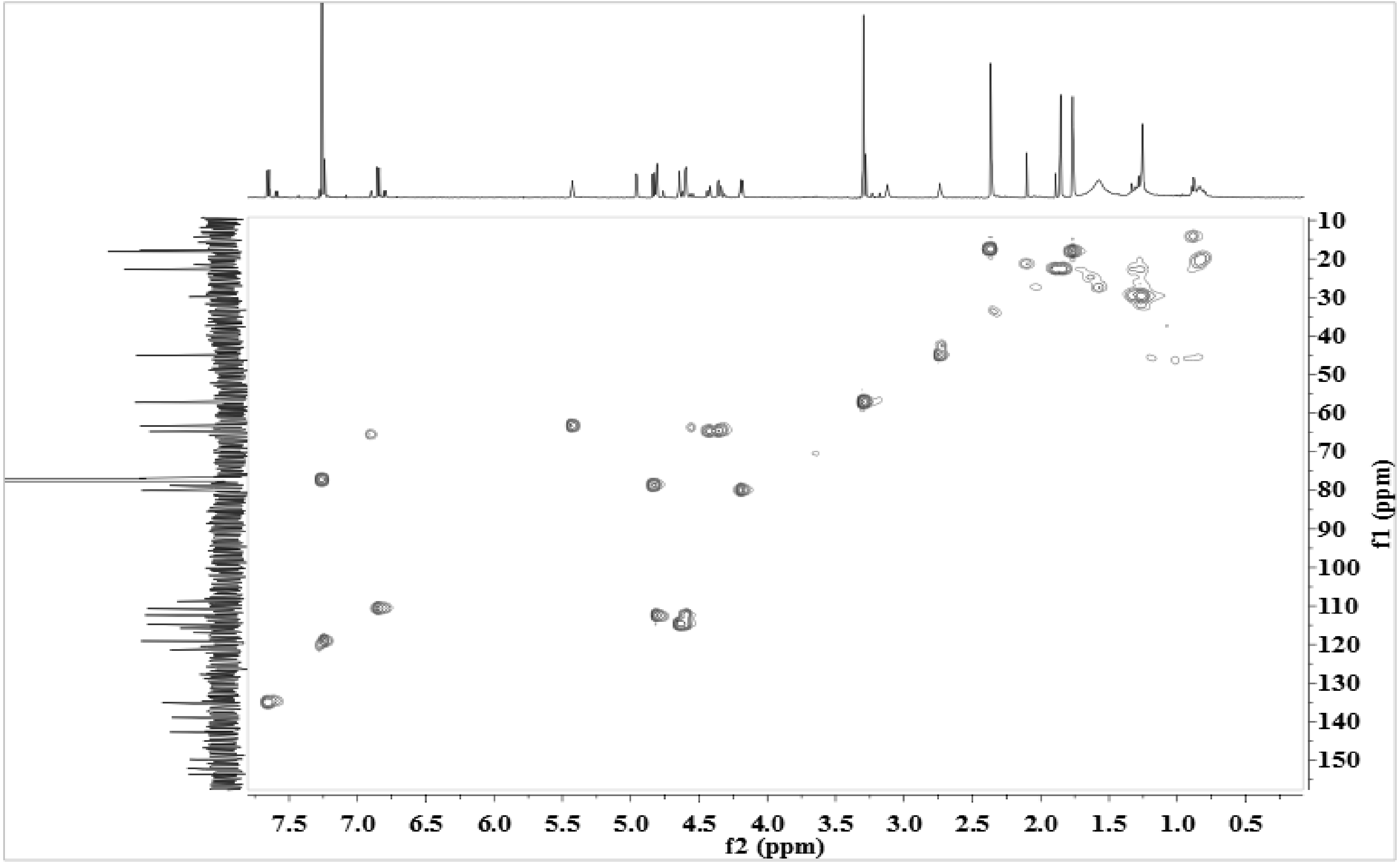

Figure S20. HSQC ($CDCl_3$) spectrum of compound **3**

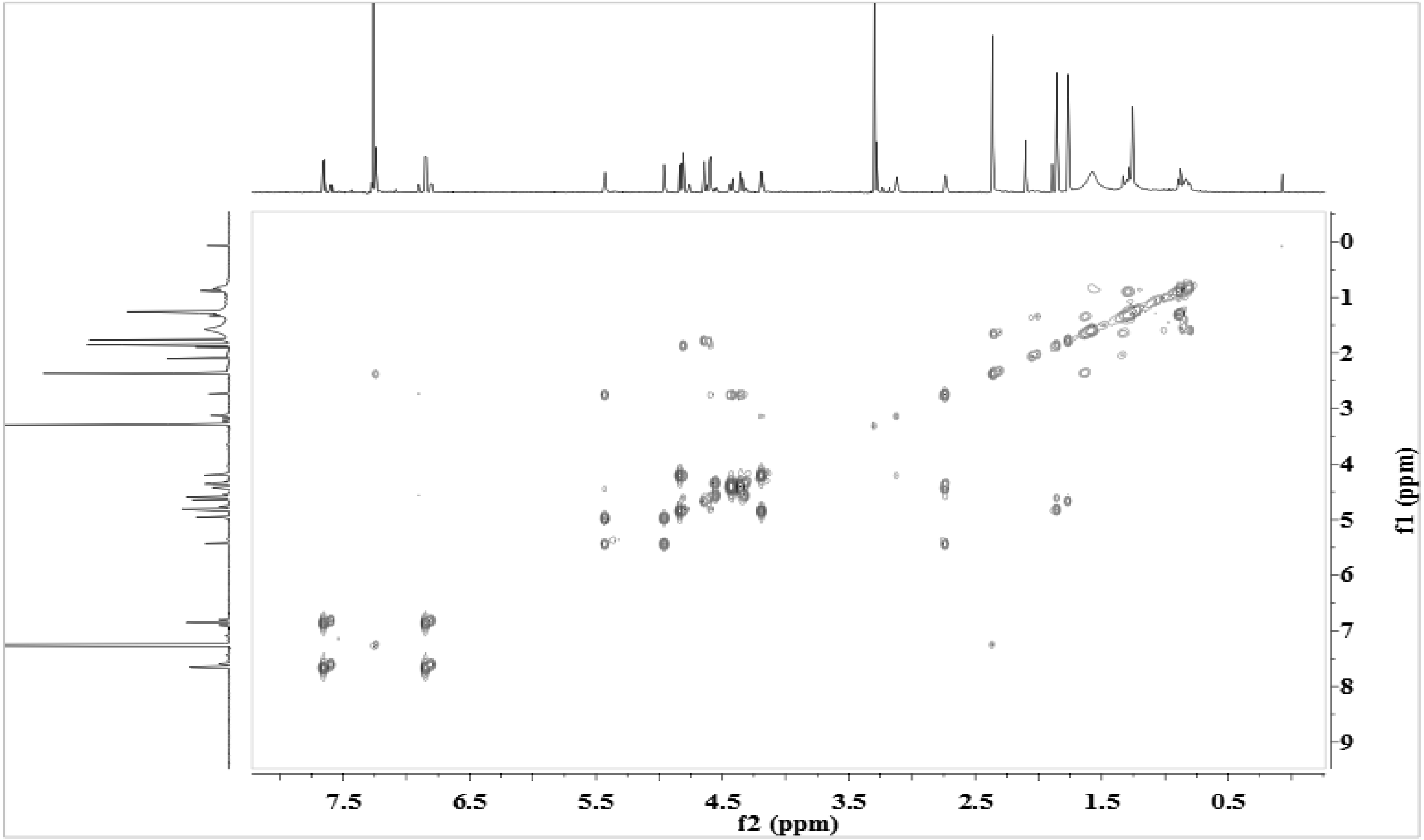

Figure S21. ^{1}H-^{1}H COSY ($CDCl_3$) spectrum of compound **3**

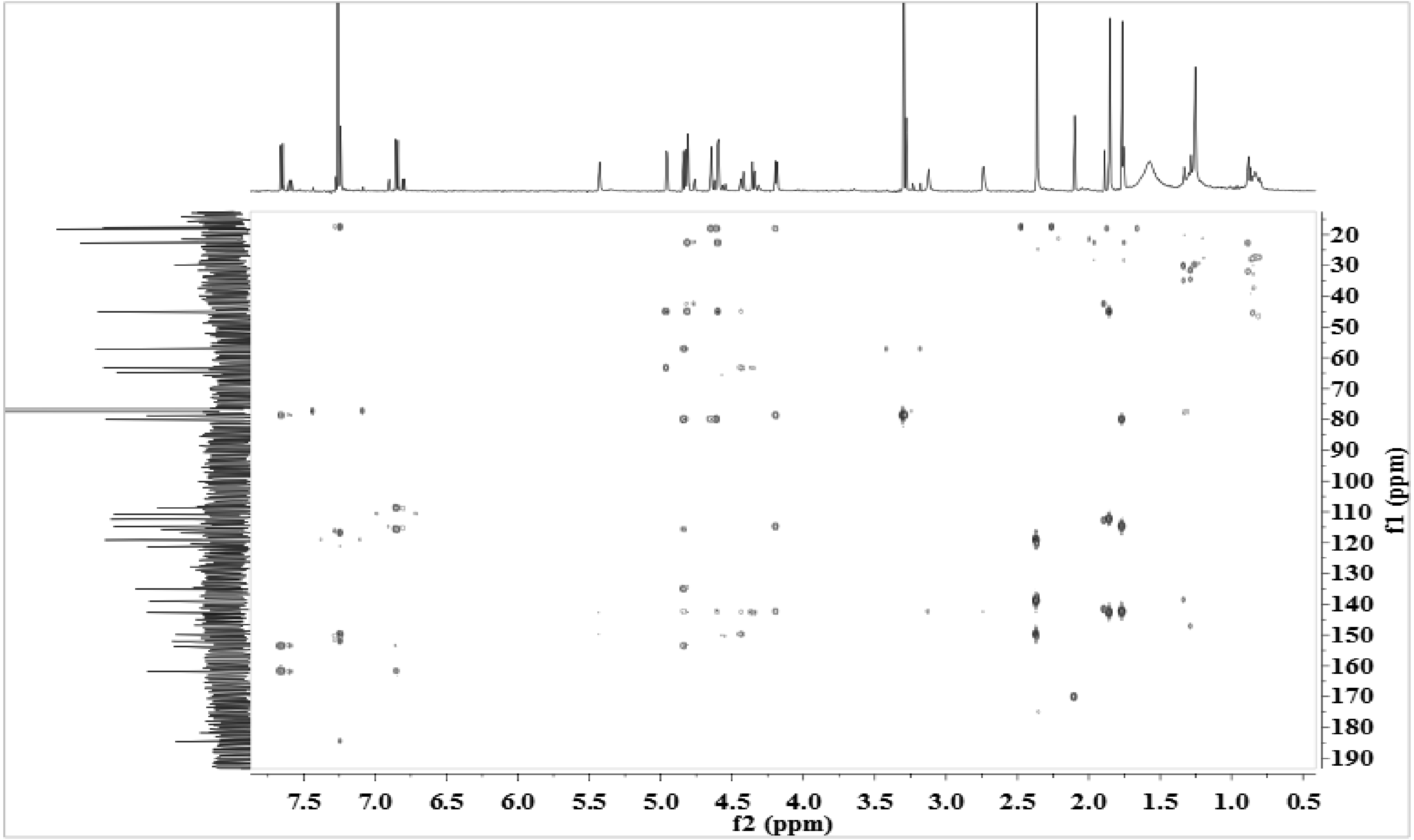

Figure S22. HMBC ($CDCl_3$) spectrum of compound **3**

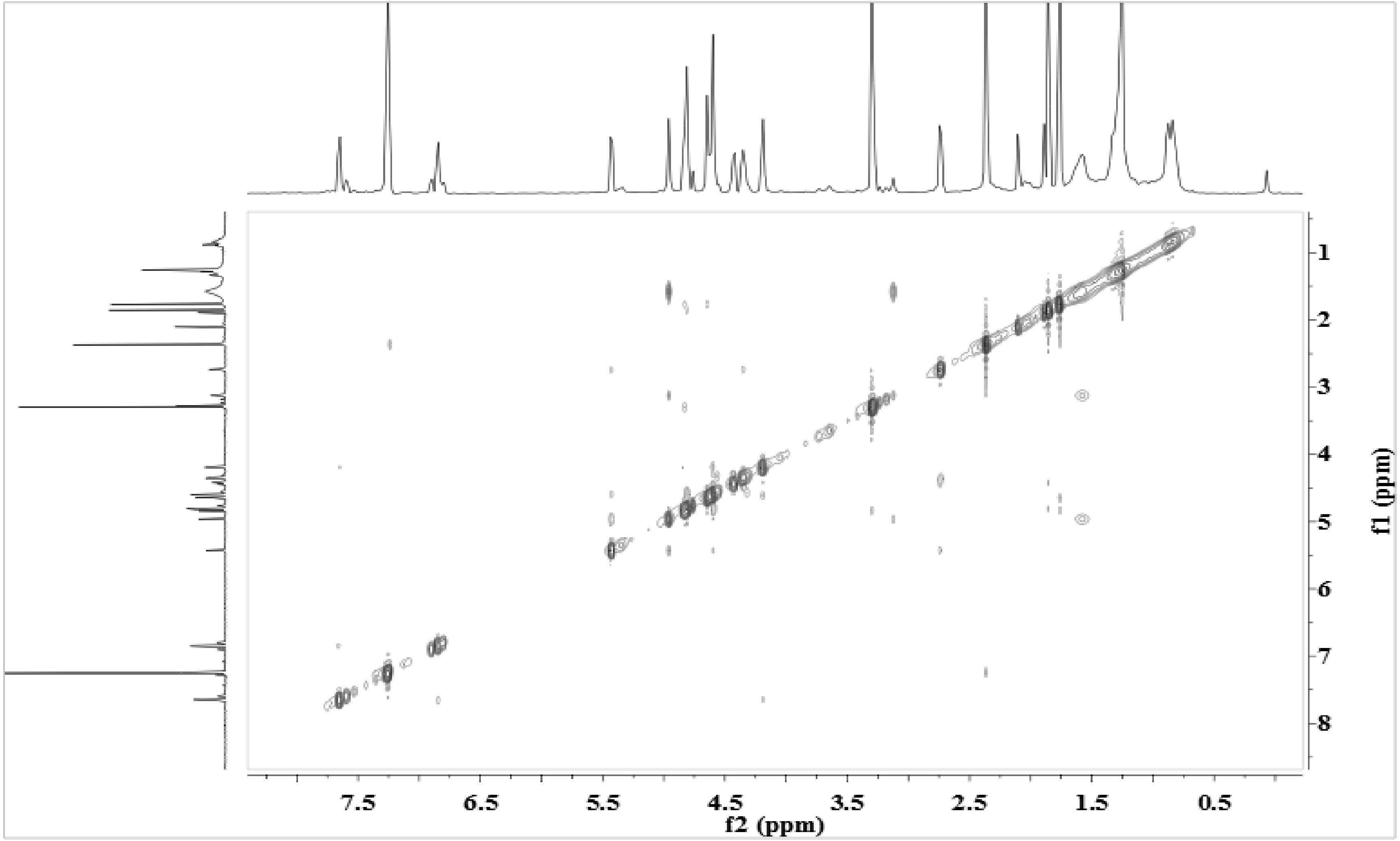

Figure S23. NOESY ($CDCl_3$) spectrum of compound **3**

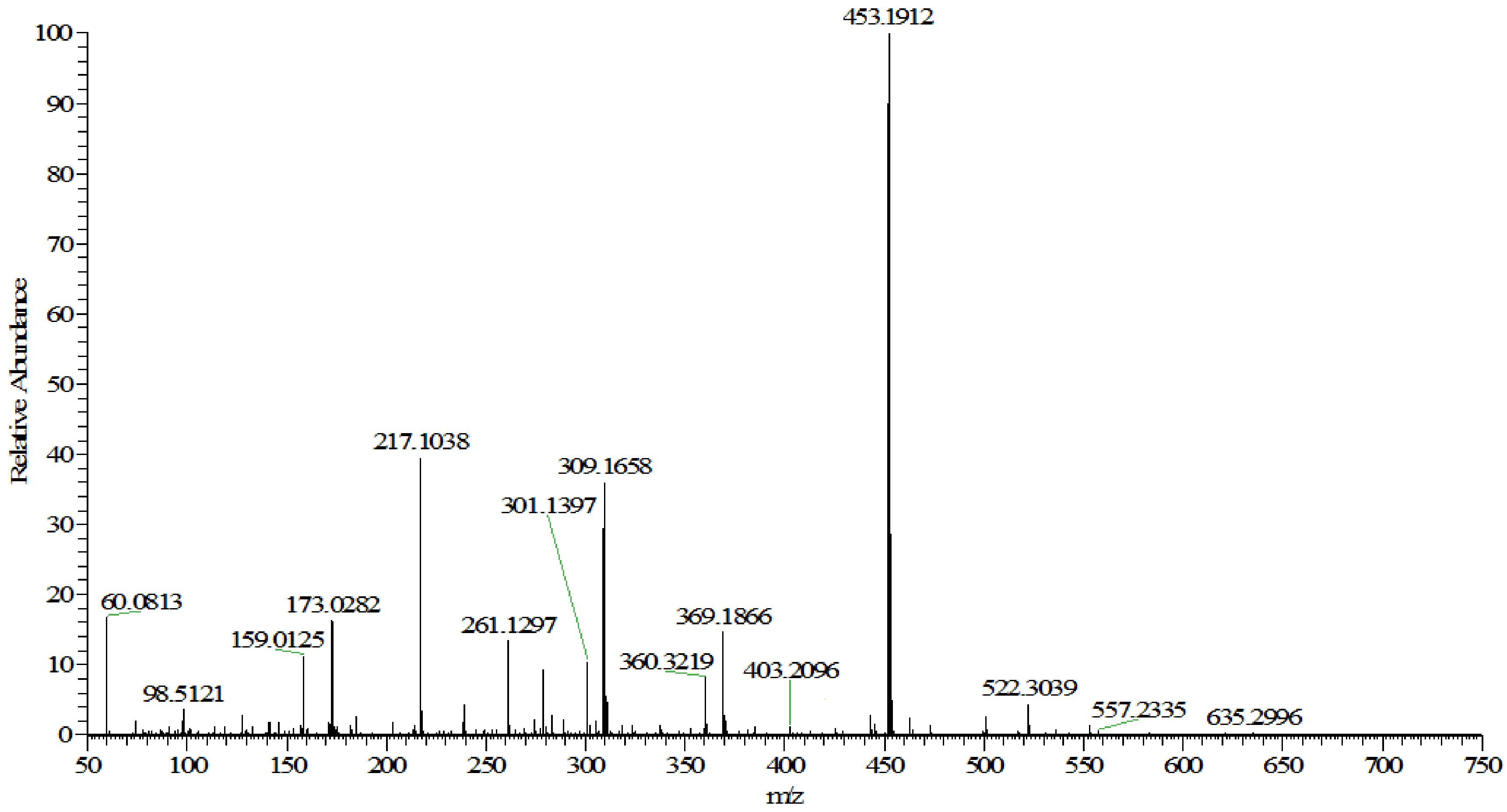

Figure S24. HRESIMS spectrum of compound **3**
